# Dual Inhibitory Activity of Petroselinic Acid Enriched in Fennel Against *Porphyromonas gingivalis*

**DOI:** 10.3389/fmicb.2022.816047

**Published:** 2022-05-19

**Authors:** Nanami Yoshino, Tsuyoshi Ikeda, Ryoma Nakao

**Affiliations:** ^1^Department of Bacteriology, National Institute of Infectious Diseases, Tokyo, Japan; ^2^Research and Analysis Center, S&B Foods Inc., Tokyo, Japan; ^3^Department of Pharmaceutical Sciences, Sojo University, Kumamoto, Japan

**Keywords:** *Porphyromonas gingivalis*, periodontal disease, gingipains, RagA/RagB, fennel, petroselinic acid

## Abstract

Increasing evidence has shown that a major periodontal pathobiont, *Porphyromonas gingivalis*, triggers oral dysbiosis leading to deterioration not only of periodontal health, but also of several systemic conditions. In the present study we identified remarkable anti-*P. gingivalis* activity of *Foeniculum vulgare* (fennel), an herbal plant used in Asian cuisine as well as in traditional medicine, by screening of 92 extracts prepared from 23 edible plants. The n-hexane-extracted fennel (HEF) showed a rapid lethal action toward *P. gingivalis*, while it was rather ineffective with a wide range of other oral commensal bacterial species. Morphological analysis using both high-speed atomic force microscopy and field emission scanning electron microscopy revealed that a low concentration of HEF (8 μg/mL) resulted in formation of protruding nanostructures composed of outer membrane vesicle (OMV)-like particles, while a high concentration of HEF (64 μg/mL) induced bacteriolysis with overproduction of OMVs with unusual surface properties. Interestingly, HEF treatment resulted in deprivation of two outer membrane transporter proteins, RagA and RagB, which is essential for nutrient acquisition in *P. gingivalis*, by extracellularly releasing RagA/RagB-enriched OMVs. Furthermore, HEF showed gingipain-inhibitory activity toward both arginine-specific (Rgps) and lysine-specific (Kgp) gingipains, resulting in blocking oral epithelial cell rounding and the subsequent detachment from culture dishes. Finally, we isolated petroselinic acid as a major bactericide as well as a gingipain inhibitor through a bioassay-guided fractionation of HEF. Taken together, our findings suggest clinical applicability of HEF and petroselinic acid for periodontitis therapy to eliminate *P. gingivalis* and its major virulence factors on the basis of the dual anti-*P. gingivalis* activity, i.e., rapid bacteriolysis and gingipain inhibition.

## Introduction

Periodontitis is a chronic multifactorial inflammatory disease associated with dysbiotic biofilms in periodontal pockets, resulting in periodontal attachment and bone loss. In a study of the global burden of oral health conducted from 1990 to 2017, the age-standardized prevalence of severe periodontitis was 9.8%, while the number of prevalent cases was estimated to be 0.8 billion (GBD 2017 Oral Disorders, 2020). In addition, emerging evidence has demonstrated epidemiological associations between periodontitis and a wide range of systemic conditions, including diabetes mellitus, preterm birth, cardiovascular disease, respiratory disease, rheumatoid arthritis, cognitive disorder, and cancer (Genco and Sanz, 2020; Genco et al., 2020; Kamer et al., 2020; Loos and Van Dyke, 2020; Nwizu et al., 2020; Orlandi et al., 2020). Therefore, periodontitis represents a significant healthcare burden on the patients worldwide.

*Porphyromonas gingivalis* is a black-pigmented Gram-negative anaerobe that resides in periodontal pockets. This microorganism is regarded as an etiological agent of periodontitis as well as dysbiotic bacterium in the oral cavity, as it disrupts the integrity of periodontal immunity by releasing a diverse repertoire of virulence factors (Lamont and Hajishengallis, 2015; Hajishengallis and Lamont, 2021). *P. gingivalis*-mediated proteolysis is known as a major pathogenic activity. Its proteolytic action is mostly dependent on the potent cysteine endopeptidase activity of gingipains (Potempa et al., 1997; Shi et al., 1999), resulting in destruction of host defense and immune systems (Hajishengallis et al., 2012; Farrugia et al., 2020), and nutrient acquisition; amino acids, oligo peptides, and heme groups; by degrading extracellular protein substrates (Kadowaki, 2021), as well as maturation of the major fimbrilin FimA by enzymatic processing (Kadowaki et al., 1998). *P. gingivalis* possesses two types of gingipains, arginine-specific (Rgps) and lysine-specific (Kgp) gingipains, which are localized on the bacterial surface, as well as extracellularly released both as free enzymes and via outer membrane vesicles (OMVs) (Nakao et al., 2014; Hirayama and Nakao, 2021). On the other hand, two major outer membrane proteins, RagA and RagB (Murakami et al., 2002), have recently been demonstrated to function as a dynamic selective outer-membrane oligopeptide-acquisition machine that is essential for efficient acquisition and utilization of proteinaceous nutrients by *P. gingivali*s (Madej et al., 2020). Furthermore, RagA and RagB were shown to be essential for growth of *P. gingivalis* by a study using the gene-deletion mutants (Nagano et al., 2007). Thereafter, we also reported that the amounts of RagA and RagB proteins were less in naturally occurring OMVs as compared to those in the outer membrane fraction (Bai et al., 2015).

Bioactive herbal-based medicine has garnered much attention as sources for new antimicrobial development, in the current context of reduced limited antimicrobial pipeline. *Foeniculum vulgare* (fennel) is an upright branching perennial generally grown for its aromatic leaves and seeds, which have long been used as an herb for cooking as well as in traditional medicine (National Library of Medicine, 2006; Badgujar et al., 2014). In Asian food culture, fennel seeds are also used as a mouth freshener or digestive aid after consuming a meal. It has been shown that essential oils obtained from the fennel leaves are characterized by a high anethole concentration and antimicrobial activity against Gram-positive bacteria (Senatore et al., 2013). Furthermore, another study reported that the Gram-negative food-borne pathogen, *Vibrio cholerae*, was killed by methanol-extracted sweet fennel seeds by inhibition of cholera toxin production (Chatterjee et al., 2016). However, the antimicrobial activity of fennel toward oral bacteria has not been systematically studied and mechanisms by which fennel contribute to antimicrobial activity remain unknown.

In the present study we investigated the antibacterial activity of fennel extract. The findings revealed two different inhibitory actions toward *P. gingivali*s, rapid bacteriolysis and gingipain-inhibitory activity. In addition, the major antimicrobial compound of fennel was isolated. Based on these findings, the unprecedented potential of fennel and the antimicrobial compound is discussed along with mechanistic insight into the dual antibacterial activities against *P. gingivali*s and the clinical applicability.

## Materials and Methods

### Preparation of Plant Extract Collection

Ninety-two extracts were prepared from 23 plants using the following four solvents.

(1) Water extraction

The starting materials were stirred in double-distilled water at 4°C for 24 h. Solubilized components were freeze-dried and stored at 4°C. Dried samples were dissolved and standardized at 10 mg/mL with double-distilled water before use. All samples were used at a final concentration of 100 μg/mL in a 96-well format screening assay.

(2) Hot water extraction

The starting materials were stirred in double-distilled water at 100°C for 1 h. Solubilized components were freeze-dried and stored at 4°C. Dried samples were dissolved and standardized at 10 mg/mL with double-distilled water before use. All samples were used at a final concentration of 100 μg/mL in a 96-well format screening assay.

(3) Ethanol extraction

The starting materials were dissolved with 100% ethanol by extensive shaking at 15–22°C for 15 min. Solubilized components were evaporated, dried, and stored at 4°C. Dried samples were dissolved and standardized at 10 mg/mL with dimethyl sulfoxide (DMSO) before use. All samples were used at a final concentration of 10 μg/mL in a 96-well format screening assay.

(4) n-Hexane extraction

The starting materials were dissolved with 100% n-hexane by extensive shaking at 15–22°C for 15 min. Solubilized components were evaporated, dried, and stored at 4°C. Dried samples were dissolved and standardized at 10 mg/mL with DMSO before use. All samples were used at a final concentration of 10 μg/mL in a 96-well format screening assay.

### Isolation of Antibacterial Compounds From n-Hexane Extracted Fennel

*Foeniculum vulgare* (fennel) were harvested in the Gujarat State region of India, situated between 23°13′00″ and 23°21′67″ N latitude and between 72°41′00″ and 72°68′33″ E longitude, during the rainy season from December 2014 to January 2015. A dry-powder form (4.0 g) of fennel seeds was extracted with 20 mL of n-hexane to yield 894 mg (dry weight) HEF. The procedure used for isolation of HEF-derived compounds is shown in Supplementary Figure 7. Briefly, a part of HEF (440 mg) was chromatographed on SiO_2_ (Φ 20 × 240) with n-hexane, n-hexane:EtOAc = 20:1,10:1,1:1, CHCl_3_:MeOH = 100:1, and CHCl_3_:MeOH:H_2_O = 90:10:1 to yield Fraction (Fr.) 1 (26 mg), Fr. 2 (42 mg), Fr. 3 (318 mg), and Fr. 4 (36 mg), respectively. Fr. 3 and Fr. 4 inhibited *P. gingivalis* growth, while Fr. 4 contained only a negligible mass and possibly some plastic derived from the laboratory container. Thus, we focused on Fr. 3 as the major fraction [ca. 75% (w/w) in HEF] and performed the following purification process. Fr. 3 was separated by reverse phase-high-performance liquid chromatography (μ-Bondapak C18 column, Φ25 × 100 × 2, Waters Co., Massachusetts, United States) with 70, 80, 85, 90, and 100% MeOH to yield to Fr.3-1 (10 mg), Fr. 3-2 (26 mg), Fr. 3-3 (198 mg), Fr. 3-4 (19 mg), and Fr. 3-5 (26 mg), respectively. Fr. 3-1, Fr. 3-2, and Fr. 3-3 inhibited *P. gingivalis* growth. A part of Fr. 3-3 (10 mg) was further separated by continuous preparative High Performance Liquid Chromatography (HPLC) using Cosmosil AR-II ODS (Φ10 × 200) with 90% MeOH to yield a compound (6.6 mg). The compound was identified as *cis-*6-octadecenoic acid (petroselinic acid: PA) by ^1^ H-, ^13^C-NMR (ECA 500, JEOL Ltd., Tokyo, Japan) and HPLC analysis compared with an authentic sample (Supplementary Figure 9). Frs. 3-1, 2 were considered to be negligible as the minor fractions and the activity might have been dependent on leakage of PA-derived compounds. All fractions and compounds derived from HEF were dissolved in DMSO. Thus, DMSO was used as the vehicle control for HEF and HEF-derived fractions/compounds in all the following experiments.

### Bacterial Strains and Culture Conditions

Bacterial strains used in this study are listed in Table 1. Three strains of *P. gingivalis* (ATCC 33277, W50, and YH522), and one strain of *Prevotella nigrescens* (ATCC 33563) were grown in brain heart infusion (BHI) broth supplemented with hemin and menadione (HM) and on BHI-HM 5% defibrinated sheep blood agar plates (BAP). An isogenic gingipain triple mutant of *P. gingivalis* ATCC 33277, termed KDP981 (Sato et al., 2013), were also grown in BHI-HM broth and on BHI-HM BAP. One strain of *Fusobacterium nucleatum* (#20), two strains of *Aggregatibacter actinomycetemcomitans* (Y4 and ATCC 29522), and nine strains of oral streptococci were grown in BHI broth and BHI-BAP. All the strains were grown in an anaerobic chamber (Whitley DG250 anaerobic workstation; Don Whitley Scientific Ltd., Bingley, United Kingdom) in 80% N_2_, 10% H_2_, and 10% CO_2_ at 37°C. A laboratory strain of *Escherichia coli*, BW25113, was also used as a model bacterium for electrophysiological analysis of the bacterial membrane. The strain BW25113 was grown in LB broth at 37°C under an aerobic condition with shaking at 150 rpm.

**TABLE 1:** MICs of n-hexane-extracted fennel seeds (HEF) and petroselinic acid (PA) against a series of oral bacteria.

	MIC (μg/mL)	
Strain	HEF	PA
*Porphyromonas gingivalis* ATCC 33277	8	5
*Porphyromonas gingivalis* W50	8	8
*Porphyromonas gingivalis* YH522	8	4
*Prevotella nigrescens* ATCC 33563	>64	>64
*Fusobacterium nucleatum* 20	>64	>64
*Aggregatibacter actinomycetemcomitans* Y4	>64	>64
*A. actinomycetemcomitans* ATCC 29522	>64	>64
*Streptococcus oralis* No.10	8	8
*Streptococcus mitis* ATCC 6245	64	64
*Streptococcus gordonii* ATCC 10588	64	64
*Streptococcus sanguinis* ATCC 10556	64	64
*Streptococcus cristatus* ATCC 51100	64	64
*Streptococcus anginosus* ATCC 33397	>64	>64
*Streptococcus salivarius* ATCC 9759	>64	>64
*Streptococcus sobrinus* ATCC 6715	>64	>64
*Streptococcus mutans* UA 159	>64	>64

### Growth Inhibition Assay and Minimum Inhibitory Concentration Determination

For screening of our plant extract collection, a growth inhibition assay was performed using *P. gingivalis* strain ATCC 33277 in a 96-well microplate (3595; Corning, New York, NY, United States) as previously described (Yoshimasu et al., 2018) with some modifications. A 48-h preculture of *P. gingivalis* was inoculated into fresh BHI-HM broth at a ratio of 1:20 (equivalent to ca. 1 × 10^8^ CFU/mL) and cultured in the presence of the water extract, hot water extract, ethanol extract, and n-hexane extract of each plant extract at final concentrations of 100, 100, 10, and 10 μg/mL, respectively. The total volume was 200 μL/well. Turbidity (absorbance at 595 nm: A_595_) of the culture broth was determined after 48-h of incubation. The presence of a growth inhibitory effect in each sample was determined when the A_595_ was less than 0.05 in the wells of the tested sample, while *P. gingivalis* cells grew normally in the vehicle control wells. A broth microdilution method was used to determine minimum inhibitory concentrations (MICs) of HEF and PA against the oral bacterial strains, according to the protocol of the Clinical and Laboratory Standards Institute (CLSI 2012; 2013), with some modifications. Briefly, following the preculture, each stain was inoculated into fresh broth at a ratio of 1:20. The broth contained a twofold dilution series of HEF or PA at final concentrations ranging from 1 to 64 μg/m. For the broth microdilution assay, the concentration was restricted to 64 μg/mL, because a greater amount could not be dissolved in the water-based broth and turbidity was thus increased, which blocked judgment of bacterial growth. A growth curve was determined by measuring turbidity (A_595_) of the culture broth in a 96-well microplate at different time points for 48 h. MICs were defined as the minimum concentration of HEF or PA that restricted growth at a level of less than 0.05 at A_595_ at all measured time points up to 48 h.

### Killing Assay

A killing assay was performed to assess the bactericidal activities of HEF and PA, as previously described (Yoshimasu et al., 2018), with some modifications. *P. gingivalis* ATCC 33277 cells standardized at 1 × 10^4^ CFU/mL in PBS were treated with HEF at the following final concentrations: 0.25, 1, 4, and 8 μg/mL; or with PA at 0.125, 0.5, and 2 μg/mL. Bacterial cells were collected and inoculated onto BHI-HM BAPs at different time points, and cultured in an anaerobic chamber for 14 days. CFUs were counted on day 14. Survival rate in the presence of HEF or PA was calculated as a relative to the CFU value for the baseline wells, in which bacteria were cultured in BHI-HM broth containing 1% DMSO (vehicle control).

### Field-Emission Scanning Electron Microscopy

*P. gingivalis* ATCC 33277 standardized at 1 × 10^8^ CFU/mL in PBS was treated with HEF at 8 or 64 μg/mL for 3 or 30 min; or with PA at 16 μg/mL for 30 min. The treated suspensions were fixed with 2.5% glutaraldehyde and 2% paraformaldehyde in PBS for 30 min, followed by three washes with PBS. After dehydration with gradually increasing concentrations of acetone, the samples were immersed in isoamyl acetate, dried by a critical point dryer, coated with osmium vapor using an osmium plasma coater, and subjected to ultra-high resolution field-emission scanning electron microscopy (FE-SEM) analysis (Regulus8220, Hitachi High-Technologies, Tokyo, Japan). The OMV particle sizes were measured using SEM images with the Fiji image processing package (a variant of ImageJ) (Schneider et al., 2012).

### High-Speed Atomic Force Microscopy

A real-time imaging system (BIXAM, Olympus Corp., Tokyo, Japan) was used to observe morphological alterations on bacterial cell surfaces at nanometer scale resolution, as previously described (Yoshimasu et al., 2018), with some modifications regarding administration of the sample to the examined bacteria (Supplementary Figure 3). Briefly, *P. gingivalis* ATCC 33277 cells standardized at 1 × 10^9^ CFU/mL with PBS were incubated on glass slides (SF17370, Matsunami Glass, Osaka, Japan) for 5 min, resulting in sufficient immobilization on the glass surface. HEF was administrated to attached *P. gingivalis* cells using a winged needle (SL-23CK, Terumo, Tokyo, Japan) connected to a glass micro-syringe (250 μL volume, 708-SNR, Hamilton, Reno, NV, United States). Sample diffusion naturally occurred. This administration system enabled observation of morphological changes without delay. Any spatiotemporal transition of the bacterial surface was continuously captured by a high-speed 3D scanner. Commercially available cantilevers BL-AC10DS-A2 (Olympus Co. Ltd.) and USC-F0.8-k0.1 (Nanoworld AG, Neuchâtel, Switzerland) were used for the high-speed tip scanning. The area of *P. gingivalis* cells every 30 s after treatment with 1% DMSO (vehicle control) or HEF were measured using High-Speed Atomic Force Microscopy (HS-AFM) images with the Fiji image processing package.

### Electrophysiological Analysis of Bacterial Membrane

For the assessment of the membrane potential of bacterial cells, flow cytometry analysis was performed as previously described (Yoshimasu et al., 2018). Briefly, *Escherichia coli* BW25113 cells were treated with HEF at various concentrations. The cells were then stained with both TO-PRO-3, a membrane-impermeable fluorescence dye, and DiOC_2_(3), a membrane potential indicator fluorescence dye. TO-PRO-3 was consistently used for staining bacterial cells together with DiOC_2_(3), to define cells with increased membrane permeability and discriminate them from depolarized cells. The TO-PRO-3-negative cell population was further divided into 2 subpopulations; polarized and depolarized cell populations in a two-dimensional dot plot. Data were analyzed using a FACS Canto II. All obtained data were analyzed with the FACS Diva software package (BD Biosciences Inc., Franklin Lakes, NJ, United States).

### Isolation of Outer Membrane Vesicles and Outer Membrane Fractions

*P. gingivalis* ATCC 33277 organisms were cultured for 48 h, then centrifuged at 5,000 × *g* for 30 min at 4°C. The supernatant was filtrated through a PVDF membrane with a pore size of 0.22 μm, then the flowthrough sample was further subjected to ultracentrifugation at 150,000 × *g* for 2 h at 4°C, to collect natural occurring OMVs (N-OMVs) as the resultant pellet, as previously described (Nakao et al., 2011). N-OMVs pellets were resuspended in 20 mM Tris-Cl (pH 8.0) and stored at –20°C. Furthermore, cell pellets obtained by low-speed centrifugation, as previously described, were washed and standardized at 1 × 10^9^ CFU with PBS, then separated into two batches. The batches were treated with 64 μg/mL of HEF in PBS or PBS without HEF but with 1% DMSO (vehicle control) with stirring for 30 min at 15–22°C. After treatment, the supernatants were collected by centrifugation at 5,000 × *g* for 30 min at 4°C, followed by filtration through a PVDF membrane with 0.22 μm pores. The flowthrough sample after treatment with HEF or the vehicle control was further subjected to ultracentrifugation at 150,000 × *g* for 2 h at 4°C, to collect HEF-induced OMVs (F-OMVs) or uninduced OMVs, respectively. The amount of uninduced OMVs was negligible, thus only F-OMVs pellets were resuspended with 20 mM Tris-Cl (pH 8.0) and stored at –20°C. Residual cells after treatment with HEF or the vehicle control were used for isolation of HEF-treated or untreated outer membrane (OM) fractions. OM fractions were prepared from *P. gingivalis* cells cultured as previously described (Nakao et al., 2008), with some modifications. Briefly, cells were washed with 20 mL of 20 mM Tris-Cl (pH 7.4) and suspended with 2 mL of 20 mM Tris-Cl (pH 7.4), then the cell suspensions were sonicated five times at 5 W for 1 min and twice at 7 W for 1 min on ice using a Handysonic UR-20P (TOMY SEIKO Co. Ltd., Tokyo, Japan) until the solution became transparent. In remove cell debris and unbroken cells, the suspension was centrifuged at 5,000 × *g* for 30 min at 4°C. The supernatant was collected, and then centrifuged at 17,400 × *g* for 1 h at 4°C. The pellets resuspended with 300 μL of 20 mM Tris-Cl (pH 7.4) were saved as the membrane fraction. In order to separate the inner and outer membranes, the membrane fraction was further treated with N-Lauryl sarcosine sodium salt at a final concentration of 2%, and then centrifuged at 17,400 × *g* for 1 h at 4°C. The resultant pellets resuspended with 300 μL of 20 mM Tris-Cl (pH 7.4) were used as OM fractions (see also Figure 2A).

### High Performance Liquid Chromatography Analysis

HPLC profiling was analyzed on a SIMADZU LC-20AT pump, SIMADZU RID-20A detector, Sugai U-620 column heater, and column of COSMOSIL 5C_18_ AR-II (5 μm, Φ4.6 × 250 mm, Nacalai Tasque Inc., Kyoto, Japan); Flow rate, 1.0 mL/min.; column temperature, 40°C; Eluting solvent, 90% MeOH in 0.02% trifluoroacetic acid. Fr. 3-3 and PA were dissolved in MeOH at 2. 02, 2.50 mg/mL concentration, respectively, and injected to the HPLC system (10 μL).

### Real-Time PCR Analysis

Two μL of undiluted culture supernatant of *P. gingivalis* ATCC 33277 was subjected to TaqMan-based real-time PCR assays of the *P. gingivalis* 16S rRNA gene using an ABI Prism 7500 (Thermo Fisher Scientific) with a Premix Ex Taq probe qPCR (Takara Bio Inc. Shiga, Japan), as previously described (Nakao et al., 2016), with some modifications. The 16S rRNA gene of *P. gingivalis* was chosen as the target for the real-time PCR assays with the following specific primer pair and probe sets: forward primer, 5′-TACCCATCGTCGCCTTGGT-3′; reverse primer 5′-CGGACTAAAACCGCATACACTIG-3′; and TaqMan probe, 5′-(FAM)-GCTAATGGGACGCATGCCTATCTTACAGCT-(TAMRA)-3′.

### Sodium Dodecyl Sulfate-Polyacrylamide Gel Electrophoresis and Western Blotting

OMVs and outer membrane fractions were analyzed with a standard protocol of Sodium Dodecyl Sulfate-Polyacrylamide Gel Electrophoresis (SDS-PAGE) protocol using 12.5% polyacrylamide gels. The same amount of protein was loaded into each lane, 0.4 μg/well for silver stain, or 2 μg/mL for western blotting (WB). Following SDS-PAGE, some gels were visualized with staining using silver (Cosmo. Bio. Co., Ltd., Tokyo, Japan) and others were transferred onto PVDF membranes for WB analysis. Membranes were blocked with 1.0% skim milk in PBS-T for 1 h at 15–22°C. Rabbit polyclonal antibodies against a hemin-binding protein 35 (anti-HBP35 antibody) generated by parenteral immunization with recombinant HBP35 (Abiko et al., 1990) and major outer membrane proteins (anti-RagA and anti-Rag B antibodies) generated by parenteral immunization with purified RagA and RagB from the outer membrane fraction of *P. gingivalis* (Murakami et al., 2004) were used at 1:1,000, 1:5,000, and 1:5,000 dilutions, respectively. A mouse monoclonal antibody against an anionic lipopolysaccharide of *P. gingivalis* (anti-A-LPS antibody) generated by parenteral immunization with purified A-LPS (Curtis et al., 1999) was used at a dilution of 1:1,000. The specificity of each antibody has been already confirmed in these previous studies (Abiko et al., 1990; Curtis et al., 1999; Murakami et al., 2004). Horseradish peroxidase-labeled anti-rabbit IgG and anti-mouse IgG (GE Healthcare, Buckinghamshire, United Kingdom) were used as the secondary antibodies at 1:200,000 and 1:20,000 dilutions, respectively. Chemiluminescence was developed with HRP Substrate (WBULS0100 Immobilon ECL Ultra Western HRP Substrate; Merck Ltd., Darmstadt, German) and visualized by exposure on X-ray film.

### Protease Inhibition Assay

The fluorogenic substrates, t-butyloxycarbonyl-L-valyl-L-prolyl-L-arginine-4-methylcoumaryl-7-amide (Boc-Val-Pro-Arg-MCA) for Rgps and t-butyloxycarbonyl-L-valyl-L-leucyl-L-lysine (Boc-Val-Leu-Lys-MCA) for Kgp were purchased from the Peptide Institute Inc. (Osaka, Japan). Protease inhibition analyses were performed as previously described (Sato et al., 2010; Kariu et al., 2017) with some modifications. *P. gingivalis* ATCC 33277 culture supernatant prepared from the 2-day culture was used as crude proteolytic enzymes containing Rgps and Kgp. 50 μL of HEF at various concentrations in 0.1 M Tris-Cl (pH 7.6) buffer containing 50 mM NaCl and 5 mM CaCl_2_ and 50 μL of the supernatant was mixed well and added to a 96-well black plate. After pre-incubation at 37°C for 10 min, 50 μL of 500 μM fluorogenic substrates was added to the mixture. Following incubation at 37°C, release of aminomethyl-coumarin was determined using a fluorescence spectrophotometer (Cytation 5, Bio Tek Instrument Inc., Winooski, VT, United States) with excitation at 380 nm and emission at 440 nm. Residual activity in the presence of HEF was calculated as relative protease activity as compared to activity in the absence of HEF (vehicle control; 1% DMSO).

### Cell Detachment Assay

Ca9-22, an oral squamous epithelial cell carcinoma cell line (Kimura, 1978), was maintained in RPMI1640 medium supplemented with 10% heat-inactivated fetal bovine serum at 37°C in a 5% CO_2_ incubator. The effect of the supernatant of 48-h cultured *P. gingivalis* on confluent monolayers of Ca9-22 cell was examined in 24-well culture plates. The cell monolayers were washed twice with 1 mL of Hanks’ balanced salt solution (HBSS) to wash out residual fetal bovine serum in the media. All tested solutions containing HEF or PA were prepared in HBSS containing 20% DMSO, and mixed with the bacterial culture supernatant at a ratio of 1:1 and incubated for 10 min at 37°C. 300 μL of the mixture containing 10% DMSO at the final concentration was added to the wells. The cells were incubated at 37°C in a 5% CO_2_ incubator, and changes in cell appearance at different time points for up to 30 min were monitored using an inverted-optical microscope (CKX41, Olympus, Tokyo, Japan). Morphological changes in cells after treatment with HEF or PA were also observed using a time-lapse imaging system with a bright-field optical microscope (WSL-1800-B, ATTO Co., Tokyo, Japan).

### Statistical Analysis

Statistical analysis was performed with a Mann-Whitney *U*-test or one-way analysis of variance (ANOVA), followed by Tukey’s multiple comparison test. *P*-values ≤ 0.05 were considered to indicate statistical significance.

## Results

### *Porphyromonas gingivalis* Shows High Sensitivity to n-Hexane-Extracted Fennel

**FIGURE 1:**
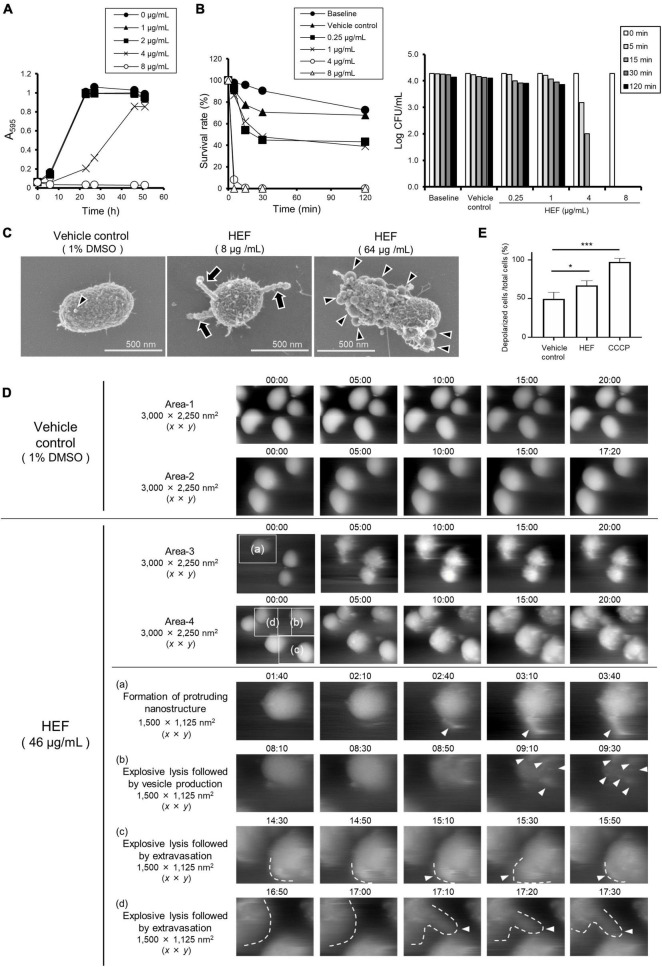
Growth, survival, and morphology of *P. gingivalis* after treatment with HEF. **(A)** Growth curves of *P. gingivalis* in the presence or absence of HEF. The bacterial cells standardized at a concentration of 1 × 10^8^ CFU/mL were treated with HEF at various concentrations. Transition of turbidity (A_595_) of the bacterial culture was monitored at different time points for 2 days. Data shown are representative of three independent experiments performed in triplicate, in which similar results were obtained. Average A_595_ values are plotted in the graph. **(B)** Killing assay to assess bactericidal activity of HEF against *P. gingivalis*. Bacterial cells standardized at a concentration of 1 × 10^4^ CFU/mL were treated without or with HEF at various concentrations for 5, 15, 30, and 120 min. *P. gingivalis* survival rate was evaluated by counting CFU on blood agar plates. For the left graph, data shown were calculated as follows: (CFU at each time point after treatment with HEF/CFU at 0 min) × 100 (%). For the right graph, the *y*-axis shows Log CFU value. Baseline indicates without treatment. Vehicle control indicates treatment with 1% DMSO as a solvent. Note that the bacterial concentration was lower than that used in the growth assay in (A). **(C)** Cell morphology observed by FE-SEM. Bacterial cells standardized at a concentration of 1 × 10^8^ CFU/mL were treated with HEF at 8 or 64 μg/mL for 30 min. Representative images are shown. Morphological changes are indicated by arrows for chain-like extrusions and arrowheads for OMVs. **(D)** Surface dynamics of *P. gingivalis* cells. Shown are HS-AFM image compilations of *P. gingivalis* following HEF treatment at a concentration of 46 μg/mL. Two randomly chosen areas sized 3,000 × 2,250 (*x* × *y*) nm^2^ treated with the vehicle control (Areas-1 and -2) or HEF (Areas-3 and -4) are shown. Images of same the area were compiled from 00:00 to 20:00. Insets denoted by (a–d) in Areas -3 and -4 of the main panels obtained at 0 min were selected, then additional images at selected time points are shown below, with higher magnification as (a–d), respectively. These areas were 1,500 × 1,125 (*x* × *y*) nm^2^. Vesicles, chain-like structures, and extravasation are denoted by white arrowheads. Cell shapes in some pictures were traced by white dotted lines. Time points after HEF treatment are indicated on the images in the format of “min:sec.” **(E)** Membrane potential (ΔΨ) analysis. *E. coli* cells were treated with HEF at a concentration of 16 μg/mL for flow cytometry analysis. Cells were also subjected to CCCP treatment as controls of depolarized cells. Data shown in the bar graph are the mean ± SD of three independent experiments. The *y*-axis shows the relative percentage of numbers of depolarized cells to total cells. *p ≤ 0.05. ***p ≤ 0.001. Statistical analysis was performed using ANOVA and Tukey’s multiple comparison test.

Ninety-two extracts were prepared from 23 plants using the following four different solvents. Screening of these extracts was performed by use of a 96-well format to examine growth inhibition activity against *P. gingivalis* ATCC 33277 strain. The results showed that only ethanol and n-hexane extracts of *Pimenta dioica* (allspice) and *Foeniculum vulgare* (fennel) completely inhibited the growth of *P. gingivalis* among the 92 plant extracts tested in this study (Supplementary Table 1). It has been already reported that allspice showed the antibacterial activity against *P. gingivalis* (Zhang and Lokeshwar, 2012; Zhang et al., 2017). However, little is known about the effect of fennel on periodontal pathogens. Thus, we here investigated how and to what extent fennel showed the antimicrobial activity against 16 oral bacterial strains including 13 different species (Table 1). In a conventional MIC determination of n-hexane-extracted fennel (HEF), *S. oralis and P. gingivalis* (MIC: 8 μg/mL) were eightfold or more than eightfold susceptible than the other oral bacterial strains (MIC: 64 μg/mL or > 64 μg/mL). Little is known about the clinical implication and its mechanism behind the high sensitivity of *S. oralis* to HEF are unclear due to the limited data. In the present study, we focused on interaction between HEF and a major periodontal pathogen *P. gingivalis* in the context of a novel therapeutic option against periodontal disease. HEF inhibited *P. gingivalis* growth in a dose-dependent manner (Figure 1A) and the growth inhibitory effect was maintained even after heat treatment at 100°C for 20 min (data not shown), while fennel extract prepared by cold or hot water as the solvent did not demonstrate any inhibition, even at 100 μg/mL (Supplementary Table 1), suggesting that the antimicrobial compound(s) in fennel are heat resistant, water insoluble, and lipophilic.

### n-Hexane-Extracted Fennel Induces Rapid Bacteriolysis With Vesicle Formation

The bactericidal activity of HEF was assessed in a standard killing assay (Figure 1B). The results showed that treatment with HEF at 4 and 8 μg/mL dramatically decreased the survival rate of *P. gingivalis* within 5 min (Figure 1B). In high-resolution FE-SEM analysis, we observed that moniliform (chain-like) protrusions composed of outer membrane vesicle (OMV)-like particles were formed on the cell surface of *P. gingivalis* following treatment with HEF at 8 μg/mL, while no change was observed in the vehicle control (1% DMSO) (Figure 1C and Supplementary Figure 1). On the other hand, a higher concentration of HEF (64 μg/mL) triggered formation of a large number of OMVs on bacterial surfaces (Figure 1C and Supplementary Figure 1) within 3 min after treatment (Supplementary Figure 2). In addition, real-time bio-imaging with HS-AFM confirmed that HEF triggered formation of moniliform nanostructures on the surface (Area-3 (a) in Figure 1D and Supplementary Movie 2A). After treatment with HEF, bacterial cells gradually swelled, which was subsequently exploded and shrunk (Supplementary Figure 4). At the same time the cells released the contents into the extracellular milieu [Area-4 (b), (c), (d) in Figure 1D and Supplementary Movie 2B], while there was no obvious change after administration of the vehicle control (Areas 1 and 2 in Figure 1D and Supplementary Movies 1A,B). In addition, HEF triggered chromosomal DNA leakage into the extracellular milieu, as the amount of extracellular DNA was approximately fivefold increased by treatment with HEF [HEF treatment (5.0 ± 0.52)^8^ vs. non-treatment (0.96 ± 0.50)^8^ copies/μL of supernatant, *P ≤ 0.05], demonstrating disruption of bacterial membrane integrity by HEF. Furthermore, an electrophysiological analysis (Yoshimasu et al., 2018) revealed that HEF induced membrane depolarization (Figure 1E and Supplementary Figure 5). Together, these findings revealed the presence of bacterial membrane-acting compound in HEF.

### n-Hexane-Extracted Fennel Deprived *Porphyromonas gingivalis* of RagA and RagB by Releasing RagA/RagB-Enriched Outer Membrane Vesicles

**FIGURE 2:**
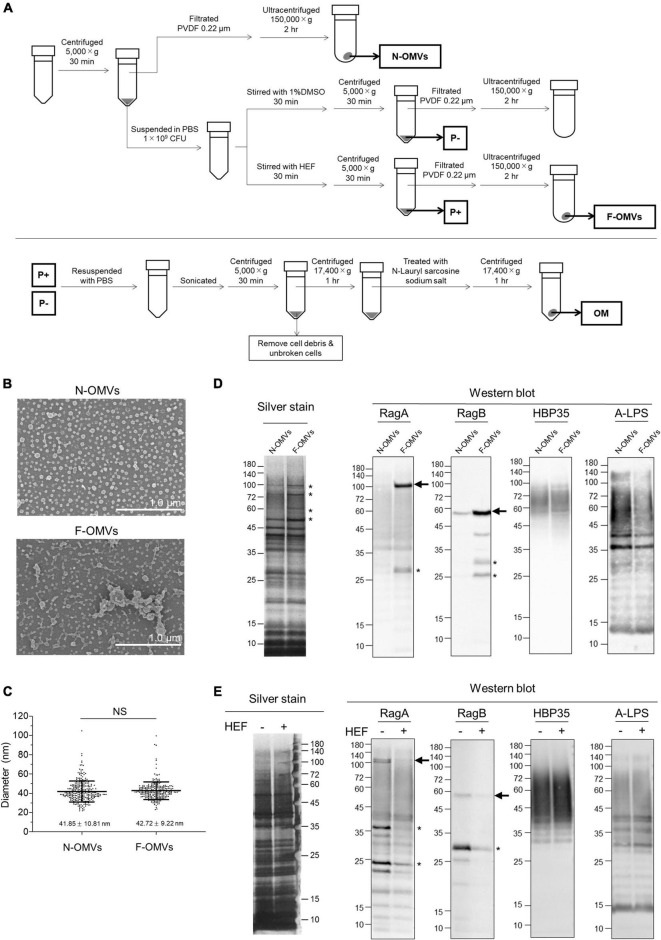
Analysis of outer membrane vesicles (OMVs) and outer membrane fraction of *P. gingivalis.* **(A)** The schematic illustration for isolation of outer membrane vesicles (OMVs) and outer membrane (OM) fractions. Naturally occurring OMVs (N-OMVs) were obtained from a 2-day bacterial culture, while HEF-induced OMVs (F-OMVs) were obtained from the supernatant of 2-day cultured bacterial cells that were pretreated with HEF at the concentration of 64 μg/mL. Outer membrane (OM) fractions prepared from *P. gingivalis* cells after treatment without or with HEF. **(B)** SEM images of N-OMVs and F-OMVs. Representative SEM images are shown. **(C)** Distribution analysis of N-OMVs and F-OMVs with diameters. Horizontal bars represent the mean ± SD. Statistical analysis was performed using a Mann-Whitney *U*-test (NS; not significant). **(D)** Silver staining and WB analysis of N-OMVs and F-OMVs. The same amount of protein (0.4 μg/well for silver staining, 2 μg/mL for WB) was loaded into each lane. Data shown are representative of three independent experiments, with similar results obtained in each. Minor differences in banding patterns between N-OMVs and F-OMVs were denoted by asterisks in the silver-stained gel. Arrows on the right of the RagA and RagB WB membranes indicates bands corresponding to the calculated molecular weights of 112,212.96 and 54,758.75, respectively, which were estimated after removal of the signal peptides. **(E)** Silver staining and WB analysis of outer membrane (OM) fractions prepared from *P. gingivalis* cells after treatment without or with HEF. The same OM fraction amount was loaded into each lane. Anti-Rag A, Anti-Rag B, Anti-HBP35, and Anti-A-LPS antibodies were used in WB analysis. Note that diffuse signals probed by Anti-HBP35 and Anti-A-LPS were due to modification with anionic polysaccharides. Asterisks denote unidentified signals, probably due to either a non-specific reaction or reaction with degraded products.

Given these morphological and electrophysiological findings, we try to characterize *P. gingivalis* OMVs that were formed by HEF treatment. There was no difference in the diameter between HEF-induced OMVs (F-OMVs) and naturally occurring OMVs (N-OMVs), while F-OMVs showed greater aggregation than N-OMVs (Figures 2B,C and Supplementary Figure 6), indicating an unusual surface property of F-OMVs. Sliver-stained gel analysis revealed that the protein profile of F-OMVs were similar to that of N-OMVs, while only minor differences were found at a high molecular weight range from 50 to 110 kDa (Figure 2D with asterisks). In WB analysis, both RagA and RagB were enriched in F-OMVs but not in N-OMVs, while there were no differences in the amounts of other outer membrane protein HBP35 or A-LPS (Figure 2D) used as two representative markers localized at outer membrane of *P. gingivalis*. We have also examined whether RagA and RagB are present in the *P. gingivalis* whole cells before and after HEF treatment. Interestingly, the amounts of both RagA and RagB in the whole cells were significantly decreased after HEF treatment. In contrast, there was no difference in the amounts of HBP35 as well as A-LPS (Figure 2E). These findings suggested that HEF deprived *P. gingivalis* of RagA and RagB by releasing RagA/RagB-enriched OMVs.

### n-Hexane-Extracted Fennel Shows Inhibitory Activity Against Gingipains

**FIGURE 3:**
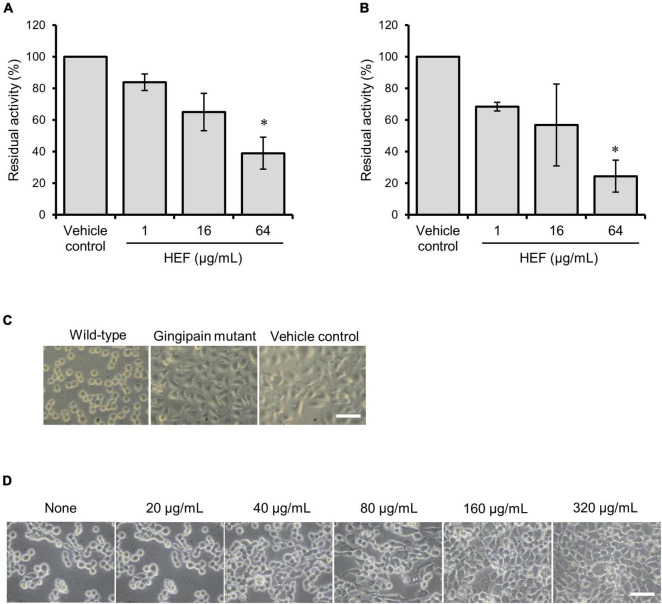
Inhibitory activity of HEF against gingipain. **(A,B)** Inhibitory effects of HEF against enzymatic activities of **(A)** Rgps and **(B)** Kgp. *P. gingivalis* wild-type strain culture supernatant was preincubated with HEF at various concentrations, then substrates for Rgps **(A)** and Kgp **(B)** were incubated with the culture supernatant that contains both Rgps and Kgp. Residual enzyme activity was measured. The value for enzymatic activity in the vehicle control (absence of HEF) was considered to be 100%. Data are expressed as the mean ± SD from results obtained in three independent experiments. *p ≤ 0.05 vs. vehicle control. Statistical analysis was performed using ANOVA and Tukey’s multiple comparison test. **(C,D)** Ca9-22 cell detachment assay **(C)** Ca9-22 cells were incubated with culture supernatants of *P. gingivalis* wild-type, gingipain triple mutant, or without culture supernatant. **(D)** Ca9-22 cells were incubated with culture supernatant of *P. gingivalis* wild-type without or with preincubation with different concentrations of HEF at 20, 40, 80, 160, and 320 μg/mL. Bars: 30 μm.

Gingipains are considered to be a cardinal virulence factor of *P. gingivalis*. HEF shows inhibitory activity against both Rgps or Kgp in a dose-dependent manner, and HEF at the concentration of 64 μg/mL significantly inhibited the enzymatic activity of both (Figure 3A, Rgps; Figure 3B, Kgp). In a cell detachment assay using oral squamous epithelial cells, the culture supernatant of *P. gingivalis* wild type strain strongly induced cell rounding and the subsequent cell detachment from the culture dishes (left column in Figure 3C and Supplementary Movies 3A,B) as compared when treated with the HBSS control (right column in Figure 3C). The action on the epithelial cells was dramatically inhibited when the culture supernatant of the isogenic gingipain triple mutant strain was used (Center column in Figure 3C). Of note, HEF protected from proteolysis by gingipains in a dose dependent manner (Figure 3D and Supplementary Movie 4A: HEF at 160 μg/mL, Supplementary Movie 4B: HEF at 640 μg/mL).

### Isolation of Bactericidal Compound From n-Hexane-Extracted Fennel

**FIGURE 4:**
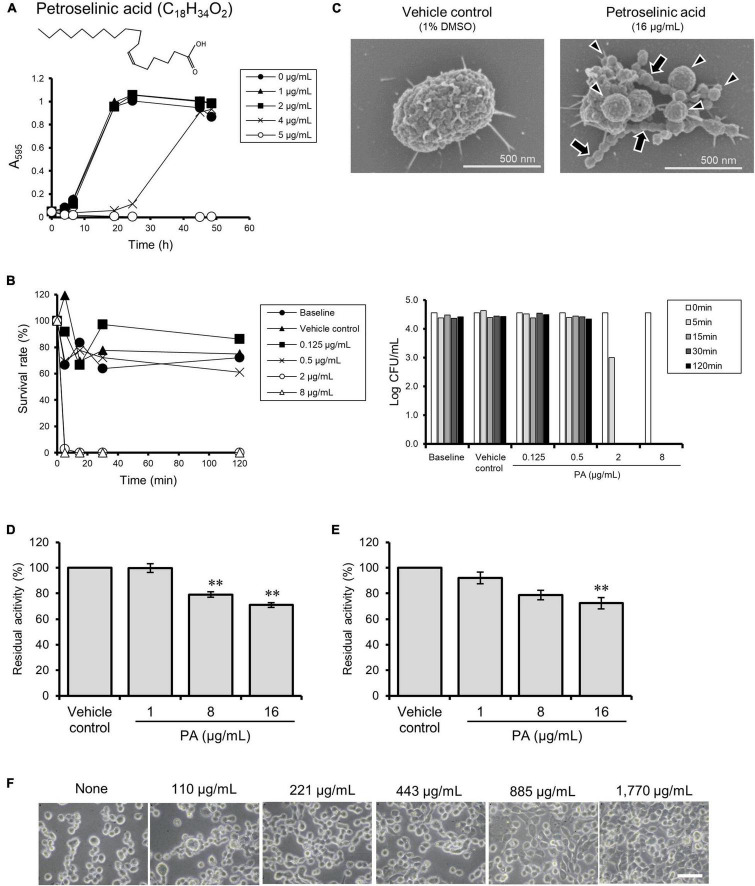
Effects of petroselinic acid (PA) on *P. gingivalis.* **(A)** Growth curves of *P. gingivalis* in presence or absence of PA. Bacterial cells standardized at a concentration of 1 × 108 CFU/mL were treated with PA at various concentrations. Transition of turbidity (A595) of the bacterial culture was monitored for 2 days at different time points. Data shown are representative of three independent experiments performed in triplicate, in which similar results were obtained. Average A595 values are plotted in the graph. **(B)** Killing assay to assess bactericidal activity against *P. gingivalis*. Bacterial cells standardized at the concentration of 1 × 10^4^ CFU/mL were treated with PA at various concentrations. The survival rate of *P. gingivalis* was evaluated by counting CFUs on blood agar plates. For the left graph, data shown were calculated as follows: (CFU at each time point after treatment with PA/CFU at 0 min) × 100 (%). For the right graph, the *y*-axis shows Log CFU value. Baseline indicates without treatment. Vehicle control indicates treatment with 1% DMSO as a solvent. Note that the bacterial concentration was lower than that used in the growth assay in **(A)**. **(C)** Cell morphology observed by SEM. Bacterial cells standardized at a concentration of 1 × 10^8^ CFU/mL were treated with PA at 16 μg/mL for 30 min. Representative images are shown. Morphological changes are indicated by arrows for chain-like extrusions and arrowheads for membrane blebbing. **(D,E)** Assay for enzymatic activity of Rgps **(D)** and Kgp **(E)**. Culture supernatant from *P. gingivalis* wild-type strain was preincubated with PA at various concentrations. Residual enzyme activity was measured. The value of enzyme activity for the vehicle control (absence of PA) was considered to be 100%. Data are expressed as the mean ± SD from results obtained in three independent experiments. **p ≤ 0.01 vs. vehicle control. Statistical analysis was performed using ANOVA and Tukey’s multiple comparison test. **(F)** Ca9-22 cell detachment assay. Ca9-22 cells were incubated with culture supernatant of *P. gingivalis* wild-type strain without or with preincubation with different concentrations of PA at 110, 221, 443, 885, and 1,770 μg/mL. Bars: 30 μm.

The antibacterial compound present in HEF was isolated with repeated fractionation and purification by use of a *P. gingivalis* growth inhibition activity test. The purification scheme and the results of the activity test are shown in Supplementary Figures 7, 8, respectively. During the purification process, trans-anethole was isolated as the principal compound of fennel, although trans-anethole did not show any inhibitory effect on *P. gingivalis* growth (data not shown). Finally, petroselinic acid (PA) was isolated and identified as a major antibacterial compound toward *P. gingivalis* with the data of H-NMR analysis (Supplementary Figure 9). The yield of PA from HEF was estimated to be an approximately 35% (w/w) and completely inhibited the growth of *P. gingivalis* at a concentration of 4–8 μg/mL (Figure 4A and Table 1), while all the other tested oral commensal except *S. oralis* showed eightfold or more than eightfold lower susceptibility to PA (Table 1). PA also showed a killing activity toward *P. gingivalis* (Figure 4B). In morphological analysis using FE-SEM, PA induced formation of OMVs and moniliform nanostructures on the surface (Figure 4C and Supplementary Figure 10), as seen in the case of HEF (Figure 1C and Supplementary Figures 1, 2). In addition, PA inhibited enzymatic activities of both Rgps and Kgp, and the inhibitory activities of PA against both gingipains were dose-dependent. PA at the concentration of 8 μg/mL or more significantly inhibited Rgps activity (Figure 4D), while PA at the concentration of 16 μg/mL significantly inhibited of Kgp activity (Figure 4E). Furthermore, PA also inhibited gingipain-dependent proteolytic activity toward Ca9-22 cells in a dose-dependent manner (Figure 4F and Supplementary Movie 5). As the PA concentration needed for blocking cell detachment was approximately 10-fold greater than the HEF concentration (Figure 3C), we assume that the anti-detachment activity exerted by HEF might not be dependent on the single compound PA. Similar results were obtained in time-lapse imaging of Ca9-22 cell morphology in the presence of PA at different concentrations (Supplementary Movie 5A: PA at 500 μg/mL, Supplementary Movie 5B: PA at 1,000 μg/mL, Supplementary Movie 5C: PA at 2,000 μg/mL).

## Discussion

In oral cavity more than 700 bacterial species form complex communities, i.e., biofilms (Aas et al., 2005). Biofilms in the subgingival pockets are formed by various bacterial species including some pathobionts rather than a single pathogenic species. Because biofilms are highly resistant to antimicrobials, therapeutic effect of antimicrobials is not satisfactory. Therefore, antimicrobial therapy against periodontal disease is regarded as a supplementary mean that may support the mechanical therapy, i.e., debridement by scaling and root planning, which is the gold standard to remove biofilms in the subgingival pockets (Hung and Douglass, 2002). However, the inappropriate use of antimicrobials in oral cavity is still performed in dentistry (Holmes et al., 2016; Gross et al., 2021). Recent studies showed that the oral cavity is considered one of major reservoirs of antimicrobial resistance genes in human body (Kearney et al., 2020; Garbacz et al., 2021). In the present study we showed that HEF and PA have a narrow-spectrum therapeutic effect against *P. gingivalis*, which is recognized as a keystone pathobiont for development of periodontal disease. Our results also showed that HEF and PA not only exhibited rapid killing activity, but also inhibited gingipains’ proteolytic activity. Given these findings, we suggest that treatment with HEF and PA will selectively eliminate *P. gingivalis* in the periodontal pockets not only by maintaining the homeostatic benefit formed by oral commensals, but also by circumventing the use of the existing antibiotics that may increase the risk of emergence and spread of antimicrobial-resistance organisms in oral cavity.

In the present study, we also examined the mechanism behind the anti-bacterial action of HEF. Firstly, we observed unusual, protruding nanostructures on the cell surfaces of *P. gingivalis* treated with HEF or PA by FE-SEM (Figures 1C, 4C and Supplementary Figures 1, 10). The protrusions were approximately 50 nm in width, which is wider than those of major and minor fimbriae of *P. gingivalis*, FimA and Mfa1, which are ca. 5 nm in width. The protruding structures were also distinguishable from flagella, common bacterial appendages (ca. ∼20 nm in width), as there is no constitutive gene encoding flagellar proteins in *P. gingivalis*. At a higher magnification, the extending nanostructures were observed as interconnected outer membrane vesicle-like chains (Figures 1C, 4C and Supplementary Figures 1, 10). Furthermore, by taking advantage of real-time HS-AFM bioimaging of bacteria at a native state, the protrusion’s dynamics was captured in a fully hydrated condition (Figure 1D and Supplementary Movie 2). Our real-time imaging analysis revealed that the protrusion structure was relatively rigid, because the structural body’s flexibility was quite low despite being exposed to flow in the fluid. Similar “protruding” bacterial appendages have been previously reported in several bacterial species, including *Bacillus subtilis* (Dubey and Ben-Yehuda, 2011), *Shewanella oneidensis* (Subramanian et al., 2018), *Franciesella novicida* (McCaig et al., 2013), and *Myxococcus xanthus* (Remis et al., 2014). For example, *B. subtilis* produces extruding nanostructures, termed bacterial nanotubes, through which cytoplasmic molecules are intercellularly exchanged (Dubey and Ben-Yehuda, 2011). *S. oneidensis* also has protruding appendages, known as bacterial nanowires, which function as a pathway of extracellular electron transfer, and are essential for element cycling and energy exchange (Subramanian et al., 2018). The formation mechanism and function of the protruding structures of *P. gingivalis* remain unknown. However, we consider that the formation may be triggered by membrane stress responses, as reported previously in a study of gut microbiota (Kintses et al., 2019). Further studies are required to better understand the formation mechanism and function of the protruding structures of *P. gingivalis*.

Zhao et al. (2019) reported that an antimicrobial mechanism behind bacterial behavior after treatment with biocides was proposed by a combinational approach of AFM-based quantitative data and the compositional profiling. In the present study, thanks to the recent advances in HS-AFM-based bioimaging system, with nanometer resolution (Yoshimasu et al., 2018), the swelling and shrinking behavior of HEF-treated *P. gingivalis* was able to quantitatively evaluate at a single cell level (Supplementary Figure 4). Additionally, after treatment with HEF or PA, numerous OMVs were also formed on bacterial surfaces (Figures 1C,D, 2B,C, 4C, Supplementary Figures 1, 2, 6, 10, and Supplementary Movie 2) by means of explosive cell lysis or bubbling cell death, which is distinguishable from the canonical mode of formation of naturally occurring OMVs, i.e., membrane blebbing (Yoshimasu et al., 2018; Toyofuku et al., 2019). By comparing between F-OMVs and N-OMVs in detail, we found that HEF deprived *P. gingivalis* cells of RagA/RagB by releasing RagA/RagB-enriched OMVs. Fischer et al. (2016) have previously reported downregulation of RagA/RagB in an unsaturated fatty acid-treated *P. gingivalis* (Fischer et al., 2016), which is in good agreement with our data. RagA/RagB is a nutrient acquisition machinery that is essential for the efficient utilization of proteinaceous nutrients by *P. gingivalis*. It could therefore be hypothesized that RagA/RagB deprivation may lead to cell death of *P. gingivalis* by starvation, while the casual relationship between the deprivation of RagA/RagB and the death of *P. gingivalis* remains unconfirmed.

In the present study, a *cis*-unsaturated fatty acid, PA, has been identified as the major antimicrobial compound in HEF against *P. gingivalis*, using a bioassay-guided fractionation. Shapiro reported that a wide range of *cis*-unsaturated fatty acids including PA had growth inhibitory effects on *P. gingivalis* (Shapiro, 1996). In addition, Fischer et al. found that an endogenous fatty acid, sapienic acid, with a chemical structure similar to PA demonstrated a rapid bactericidal activity against *P. gingivalis* (Fischer et al., 2013). Those reports are in good agreement with our findings regarding anti-*P. gingivalis* activity of PA (Figures 4A–C). We further expand the knowledge of PA-mediated anti-gingipain activity (Figures 4D–F).

## Conclusion

We investigated the antibacterial activity of HEF, and found two different important actions toward *P. gingivalis*; rapid bacteriolytic activity and high levels of gingipain-inhibitory activity. HEF was shown to have effects on bacterial surface dynamics, resulting in emergence of extruding nanostructures and overproduction of OMVs. Furthermore, the present findings also demonstrated that HEF dramatically depleted the essential RagA/RagB transport machinery in *P. gingivalis* cells by causing extracellular release of RagA/RagB-enriched OMVs. In addition, petroselinic acid was identified as the major antimicrobial compound of HEF.

## Data Availability Statement

The original contributions presented in the study are included in the article/Supplementary Material, further inquiries can be directed to the corresponding author/s.

## Author Contributions

NY and RN contributed to conception, design, data acquisition and interpretation, drafted and critically revised the manuscript. TI contributed to design, data acquisition and interpretation, and critically revised the manuscript. All authors contributed to the article and approved the submitted version.

## Conflict of Interest

NY was employed by S&B Foods Inc. The remaining authors declare that the research was conducted in the absence of any commercial or financial relationships that could be construed as a potential conflict of interest.

## Publisher’s Note

All claims expressed in this article are solely those of the authors and do not necessarily represent those of their affiliated organizations, or those of the publisher, the editors and the reviewers. Any product that may be evaluated in this article, or claim that may be made by its manufacturer, is not guaranteed or endorsed by the publisher.
