# Supplementary material for: Dual Inhibitory Activity of Petroselinic Acid Enriched in Fennel Against Porphyromonas gingivalis
Source: Front Microbiol. 2022 May 19;13:816047. doi: 10.3389/fmicb.2022.816047 (PMC9161081; doi:10.3389/fmicb.2022.816047)
Supplement: Supplementary file 1 [file Data_Sheet_1.pdf]

Botanical name			Turbidity (A_{595}) of *P. gingivalis* after 48 h			
Binomial nomenclature	Common name	Part	Water extract	Hot water extract	Ethanol extract	n-Hexane extract
Anethum graveolens	Dill	Seed	-	-	-	-
Artemisia dracunculus	Tarragon	Leaf	-	-	-	-
Capsicum annuum	Chile pepper	Fruit, Seed	-	-	-	-
Carum carvi	Caraway	Seed	-	-	-	-
Cinnamomum zeylanicum	Cinnamon	Bark	-	-	-	-
Citrus hystrix	Kaffir lime	Leaf	-	-	-	-
Coriandrum sativum	Coriander	Seed	-	-	-	-
Coriandrum sativum	Coriander	Leaf	-	-	-	-
Cuminum cyminum	Cumin	Seed	-	-	-	-
Curcuma longa	Turmeric	Root	-	-	-	-
Elettaria cardamomum	Cardamom	Seed	-	-	-	-
Foeniculum vulgare	Fennel	Seed	-	-	+	+
Illicium verum	Star anise	Fruit	-	-	-	-
Origanum majorana	Marjoram	Leaf	-	-	-	-
Origanum vulgare	Oregano	Leaf	-	-	-	-
Pimenta dioica	Allspice	Seed	-	-	+	+
Pimpinella anisum	Anise	Seed	-	-	-	-
Piper longum	Long pepper	Fruit, Seed	-	-	-	-
Piper nigrum	Black pepper	Seed	-	-	-	-
Rosmarinus officinalis	Rosemary	Leaf	-	-	-	-
Salvia officinalis	Sage	Leaf	-	-	-	-
Trigonella foenum-graecum	Fenugreek	Seed	-	-	-	-
Zingiber officinale	Ginger	Root	-	-	-	-

Supplementary Table 1. Effect of plant extracts on *Porphyromonas gingivalis* ATCC 33277.

Nineteen-two samples extracted from 23 raw plant by using four different solvents (water, hot water, ethanol or n-hexane) were subjected to the growth inhibition assay using *P. gingivalis* ATCC 33277. *P. gingivalis* was cultured in the presence of water extract, hot water extract, ethanol extract or n-hexane extract at the final concentrations of 100, 100, 10, and 10 µg/mL, respectively. Turbidity of bacterial culture (absorbance at 595 nm: A_{595}) was measured after incubation for 48 h to determine whether each extract shows inhibitory effect on *P. gingivalis* (+: less than 0.05 at A_{595}) or not (-: Not less than 0.05 at A_{595}).

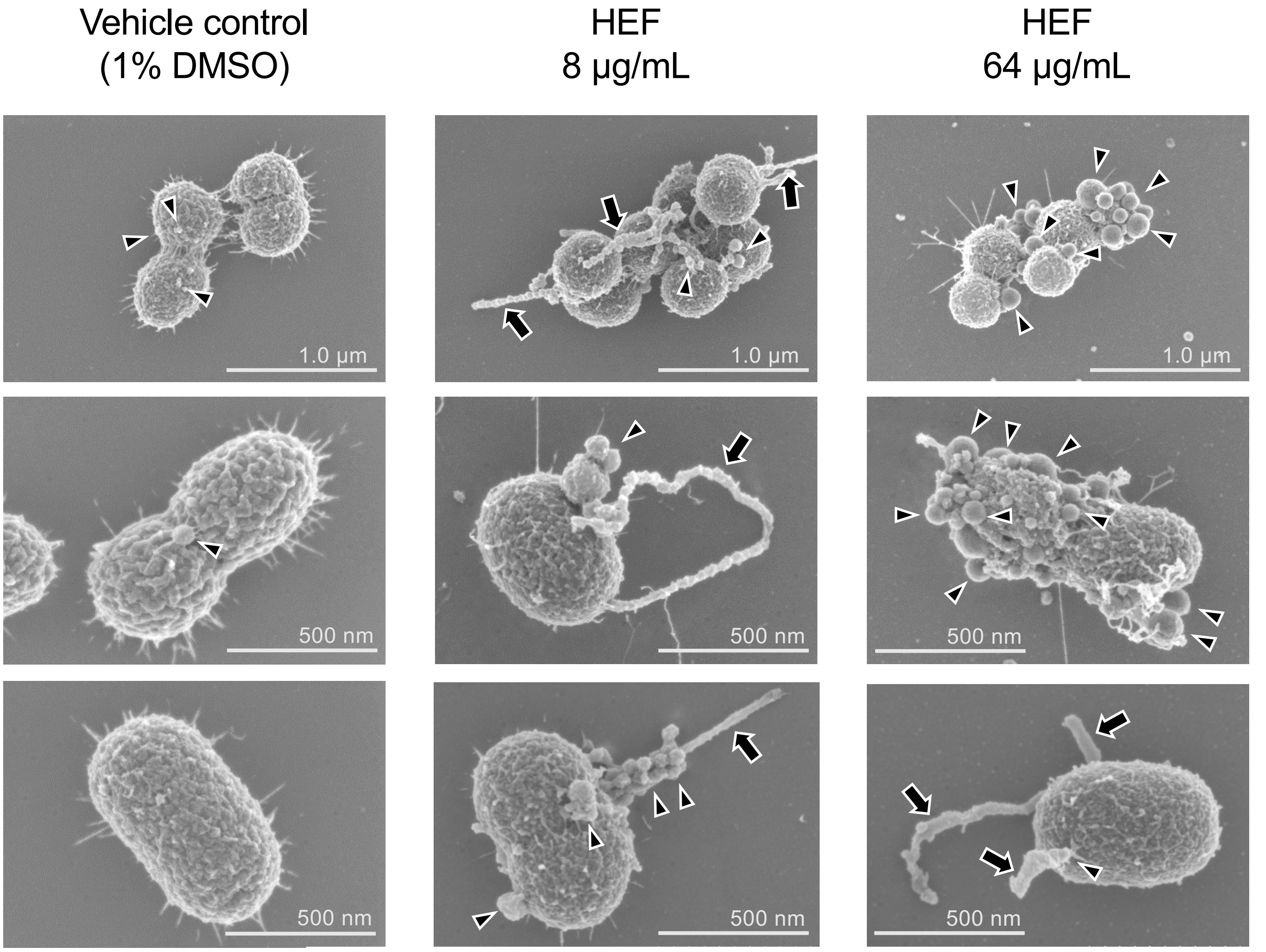

Supplementary Figure 1. Morphological observation of *P. gingivalis* ATCC 33277 treated without or with HEF.

Bacterial cells were treated with HEF at different concentrations of 0, 8 or 64 μg/mL for 30 minutes. Representative 3 SEM images are shown. Arrows indicate chain-like clusters composed of outer membrane vesicles (OMVs) formed most frequently when 8 μg/mL of HEF was treated. OMVs are also indicated by arrowheads.

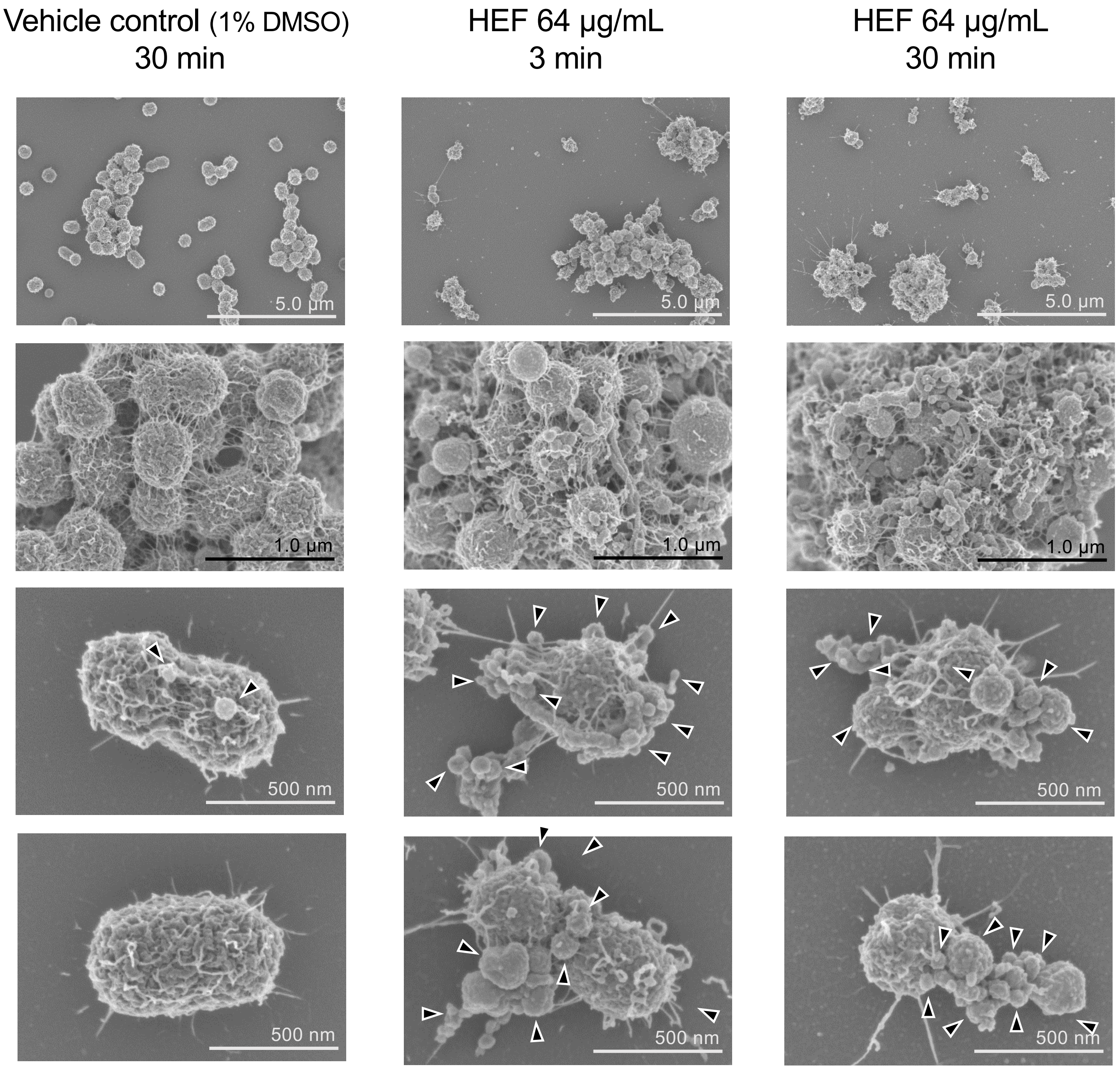

Supplementary Figure 2. Morphological observation of *P. gingivalis* ATCC 33277 treated with HEF for different times.

Bacterial cells treated with HEF at the concentration of 64 µg/mL for 3 or 30 minutes are shown in center or right columns, respectively. Bacterial cells treated with vehicle control (1% DMSO) for 30 minutes were also shown in left columns. Representative SEM images captured at different magnifications are shown. OMVs are indicated by arrowheads.

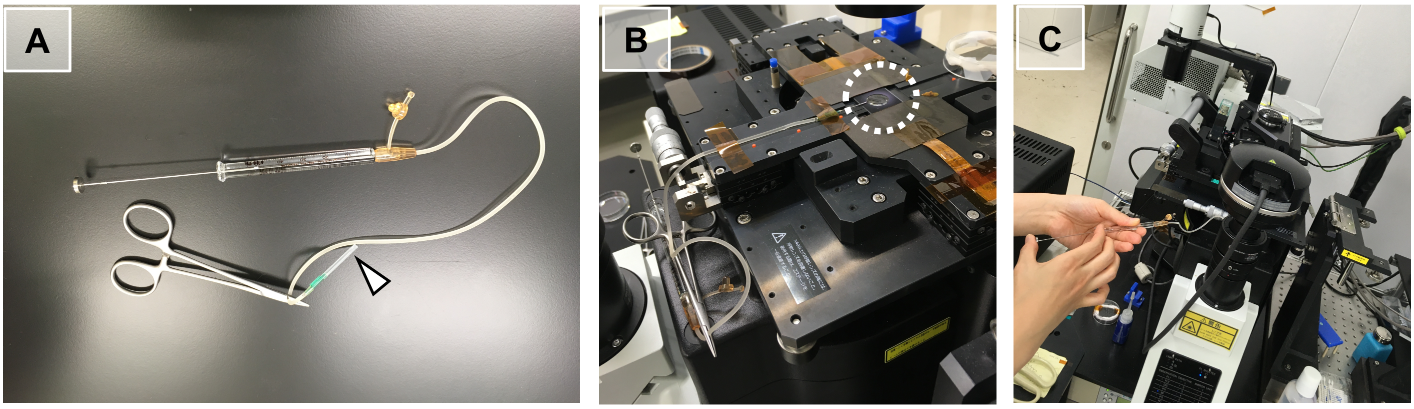

Supplementary Figure 3. Administration of sample in HS-AFM real-time imaging system.

(A) Configurations of administration/perfusion system for HS-AFM. Micro-syringe is connected with the tube with 23G wing needle (denoted by an arrowhead). The both wings were cut before use. The hemostatic forceps are also used for clamping.
(B) Installation of the system on the stage of HS-AFM. The sample to be examined is filled in the needle, tube, and micro-syringe without bubbles. On the other hand, the bacterial cells immobilized on the slide glass are covered with 50 μL of PBS. Then, the tip of the needle is set up on the bacterial samples on the slide glass placed on the stage (denoted by a dotted white circle).
(C) After the cantilever is placed on an appropriate position, the sample is manually administrated to the bacterial cells at a speed of approximately 1 μL /sec. via the tube. The sample gradually reaches to bacterial cells by natural diffusion.

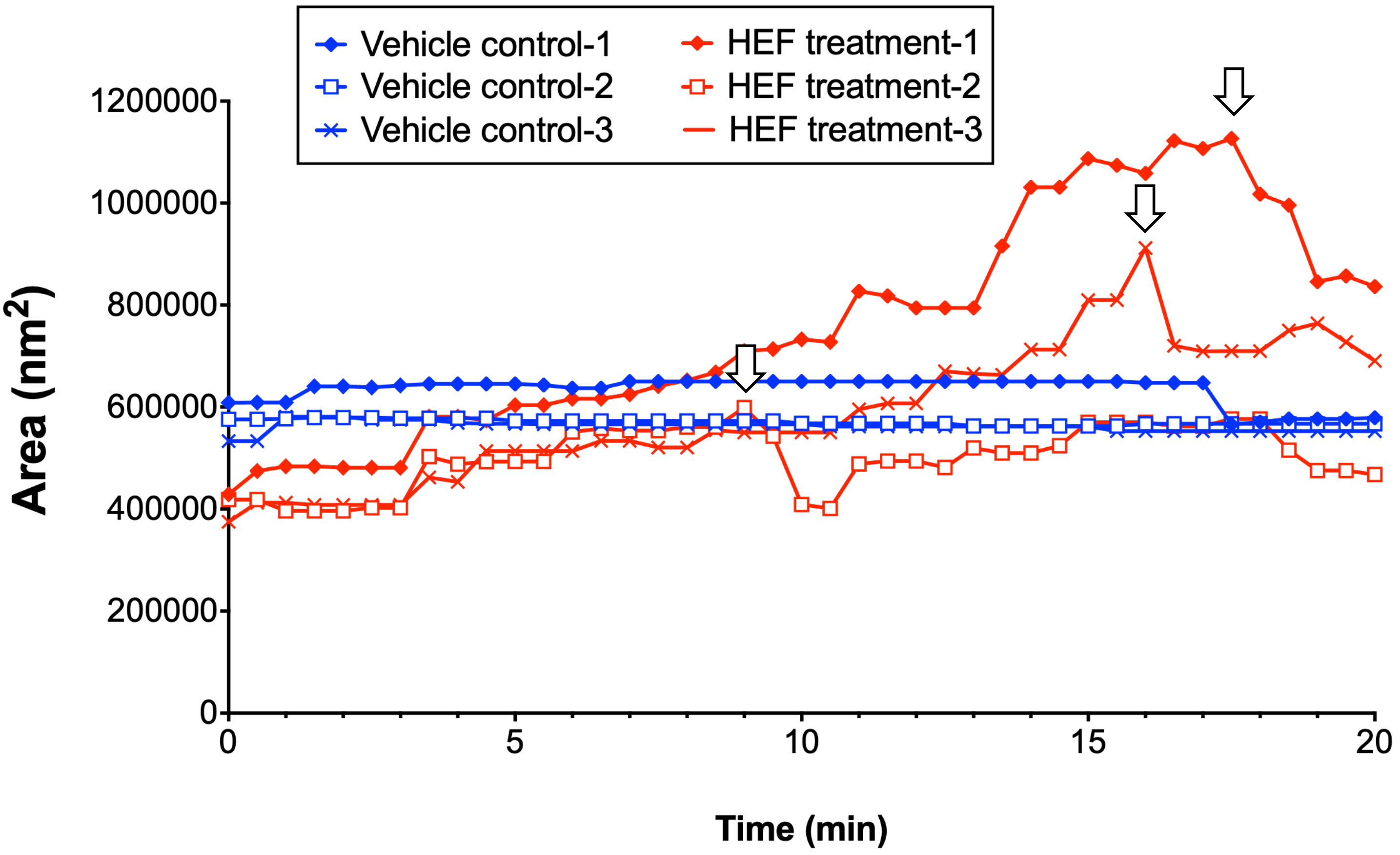

Supplementary Figure 4. The volume transition of *P. gingivalis* cells after treatment with PA.

The area of *P. gingivalis* cells every 30 seconds after treatment with 1% DMSO (vehicle control) or 46 µg/mL HEF were measured using HS-AFM images with the Fiji image processing package and plotted. For analysis three random cells were chosen, respectively. Arrows show the time point when HEF-treated-cells were exploded.

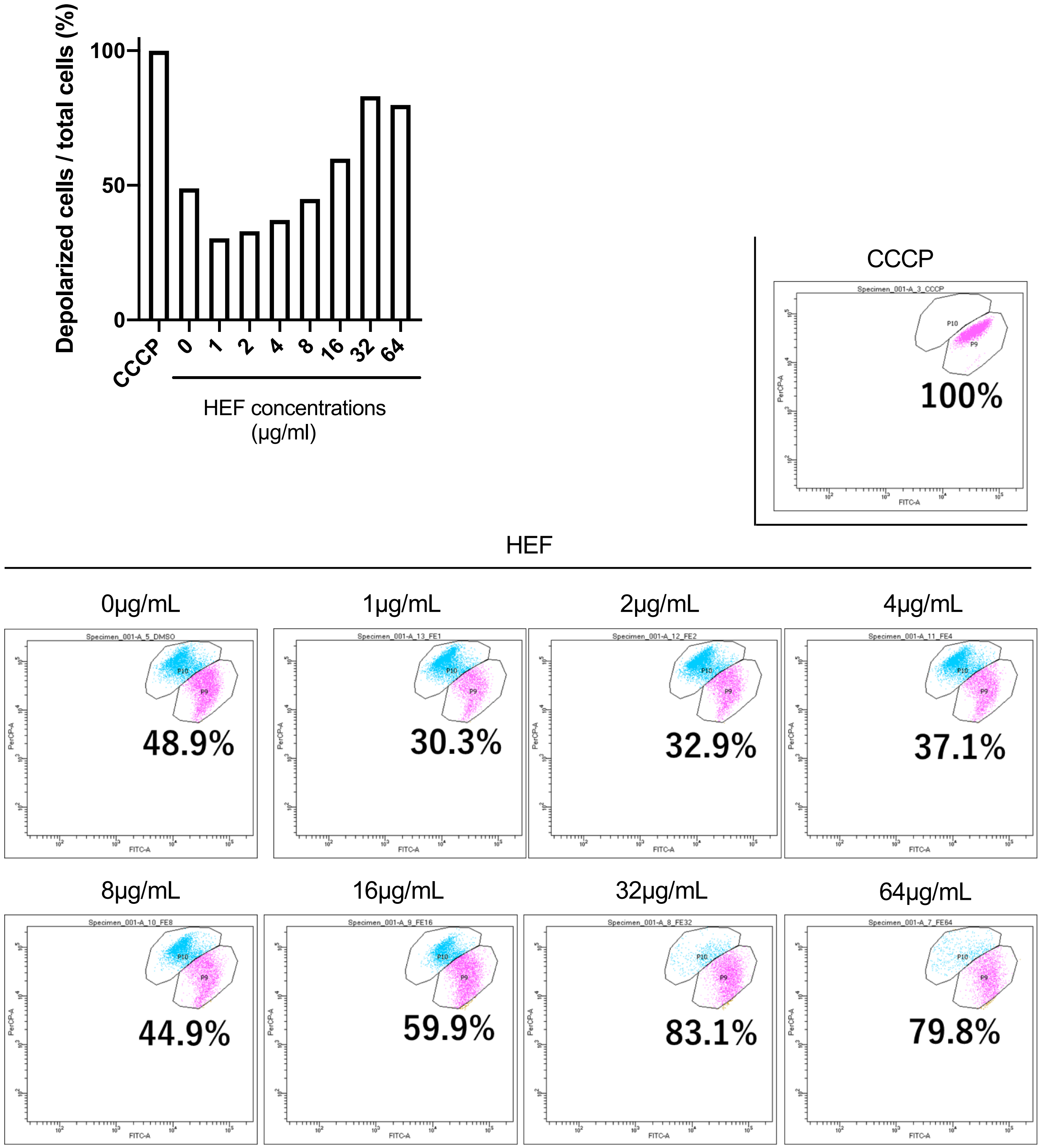

Supplementary Figure 5. Membrane potential (ΔΨ) of bacterial cells treated with HEF.

E. coli cells were treated with HEF at the different concentrations for fluorescence-activated cell sorting analysis. Cells were also subjected to CCCP treatment as controls of depolarized cells. Data shown in the bar graph at the upper left are representative of 3 independent experiments. The y-axis shows the relative percentage of depolarized cell numbers to total cell numbers.
Raw FACS data are also shown at the bottom with the percentage of depolarized cells in the total cells. In each of two-dimensional dot plots, polarized- and depolarized-cell populations are shown in sky blue and magenta, respectively. A representative data from 3 independent experiments are presented. Similar results were obtained in all the experiments.

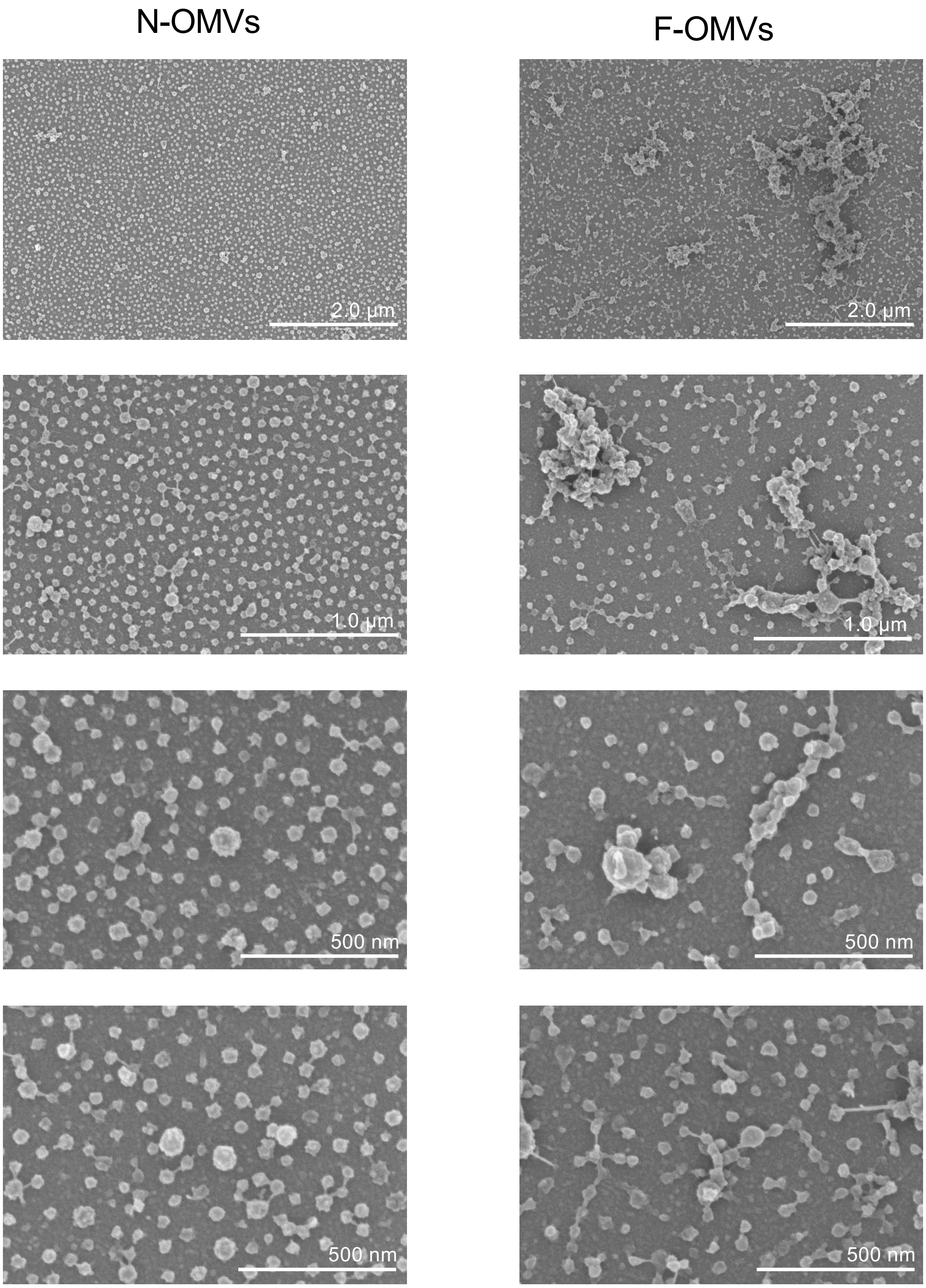

Supplementary Figure 6. Morphological observation of outer membrane vesicles (OMVs) of *P. gingivalis* ATCC 33277.

Natural occurring OMVs (N-OMVs) were collected by a two-day bacterial culture. "HEF-induced OMVs (F-OMVs) were collected from the supernatant of 2-day cultured bacterial cells that were pretreated with HEF at the concentration of 64 µg/mL. Representative SEM images are shown.

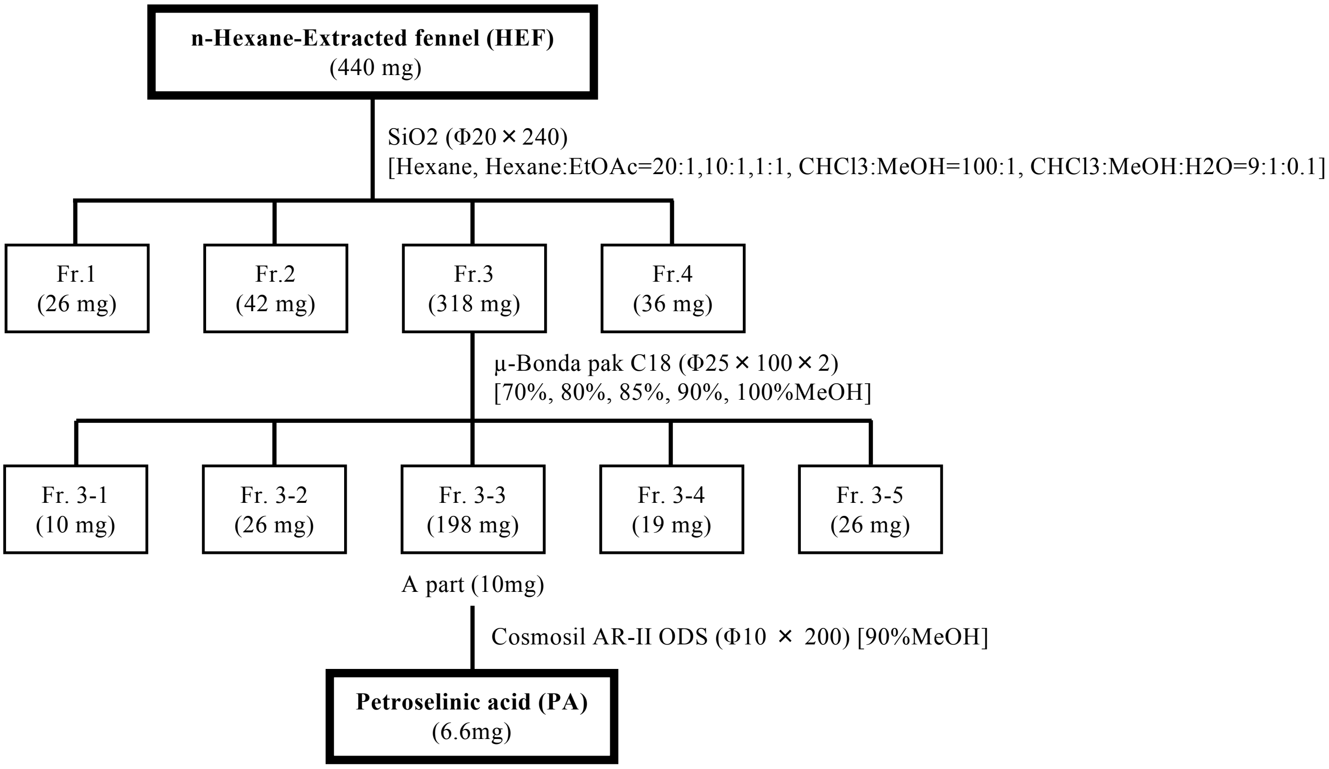

Supplementary Figure 7. Procedure for purification of bactericidal fractions from n-hexane-extracted fennel (HEF).

Overview of the purification scheme used for isolation of antibacterial compounds from HEF. At the end of purification, petroselinic acid (PA) was isolated.

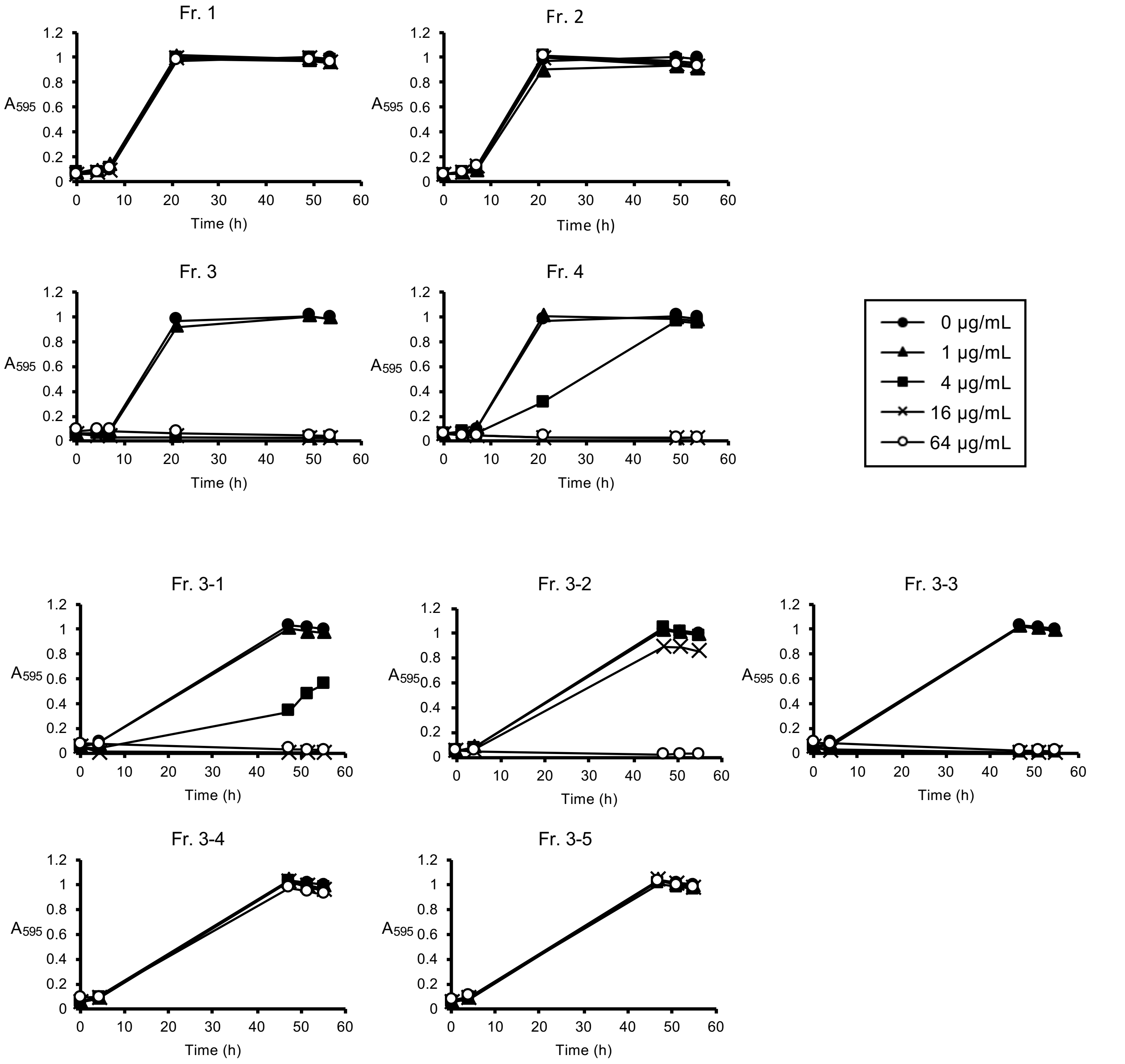

Supplementary Figure 8. Growth curves of *P. gingivalis* cells cultured in the presence of fractions prepared from HEF.

Turbidity (OD595) of the bacterial culture was monitored for 2 days at different time points. Data shown are representative of 3 independent experiments performed in triplicate. Similar results were obtained in the 3 experiments. The average of A_{595} are plotted in the graph.

A ¹H-NMR analysis

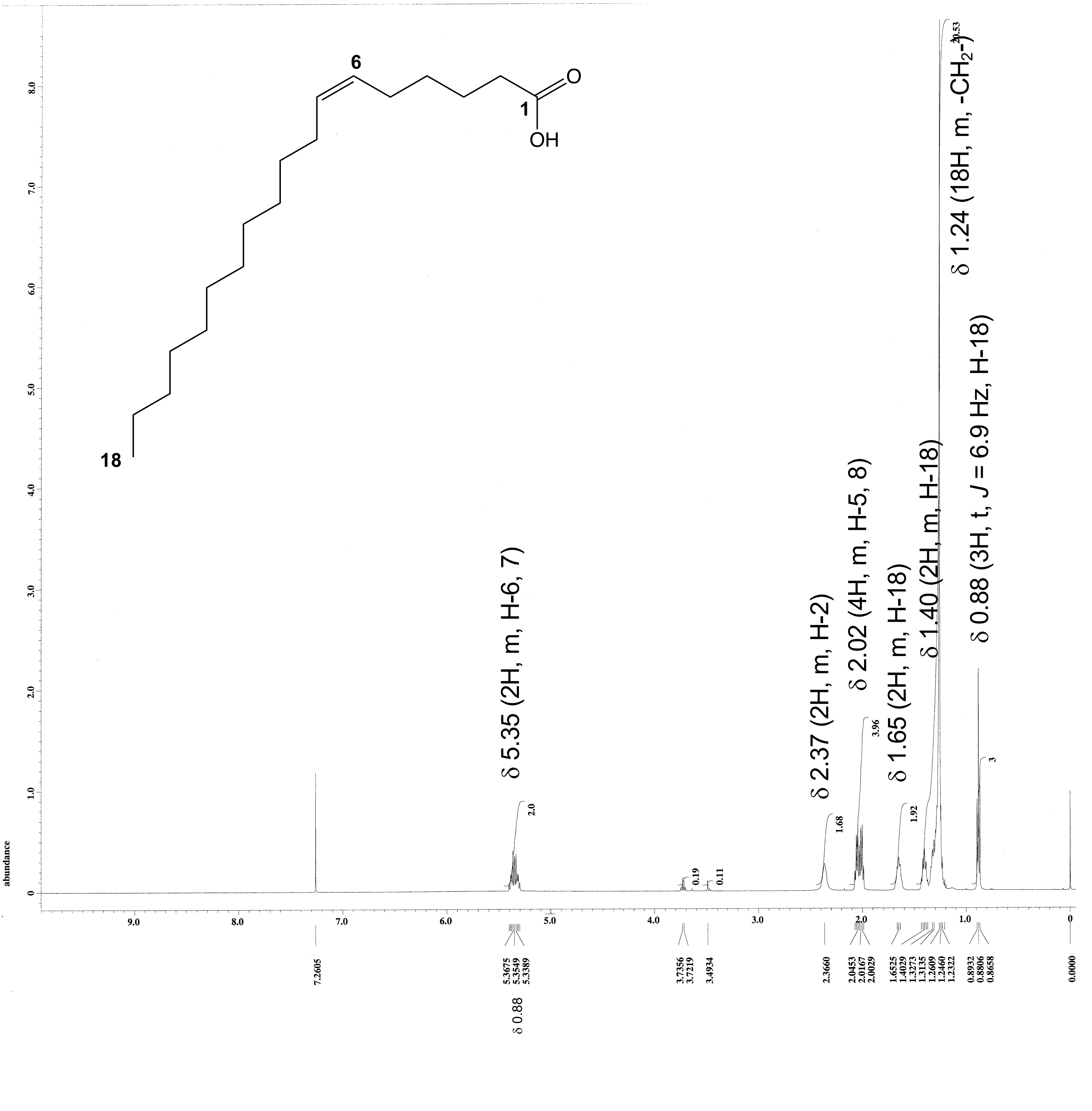

Supplementary Figure 9. Identification of PA from Fr. 3-3

(A) ¹H-NMR of *cis*-6-octadecenoic acid (petroselinic acid: **PA**) (in $CDCl_3$) isolated from Fr. 3-3. Each fraction was analyzed by ¹H-NMR (ECA 500, JEOL Ltd., Tokyo, Japan).

B ^{13}C-NMR analysis

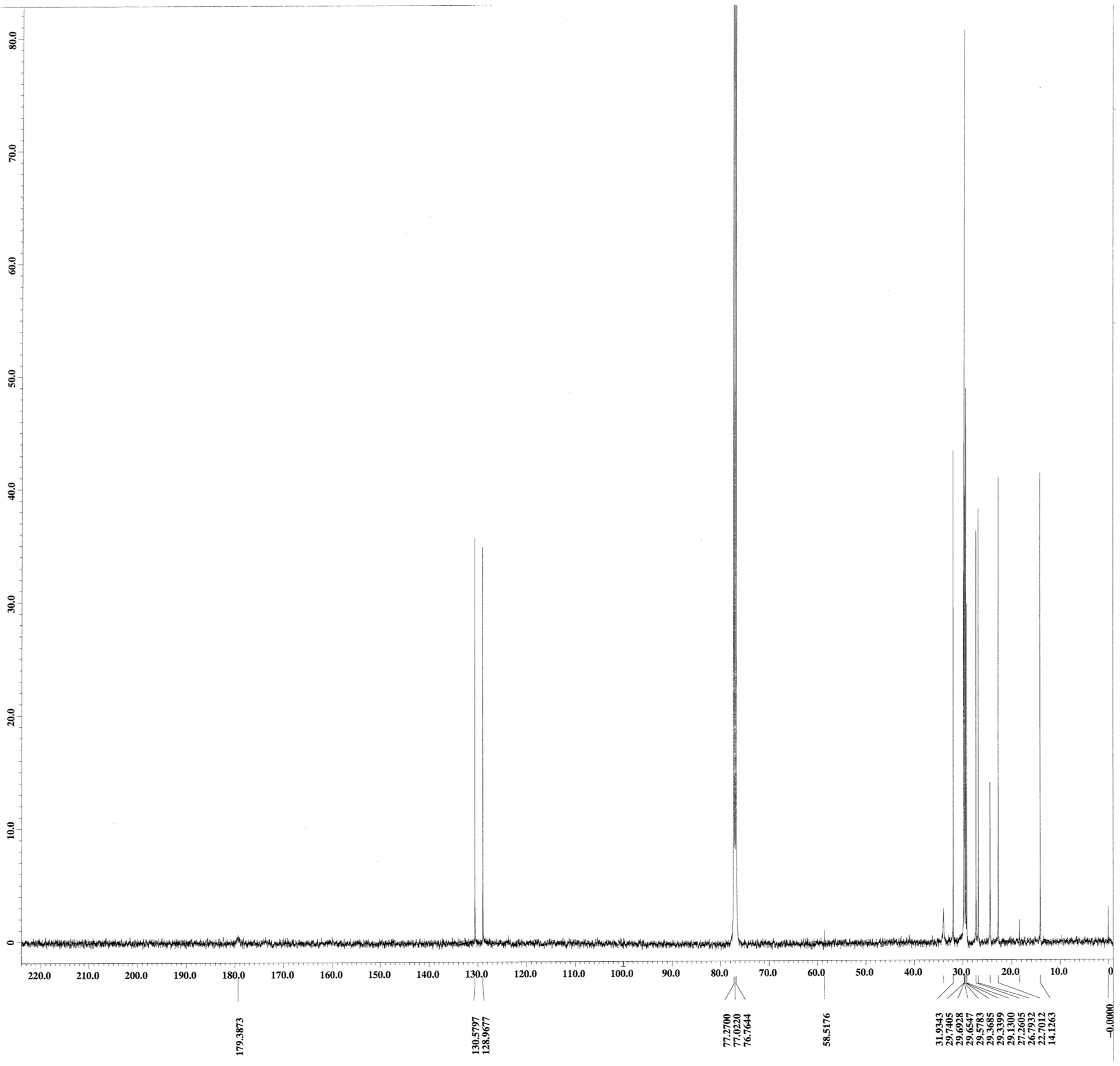

Supplementary Figure 9. Identification of PA from Fr. 3-3

(B) ^{13}C-NMR of **PA** (in $CDCl_3$) isolated from Fr. 3-3

C HPLC analysis

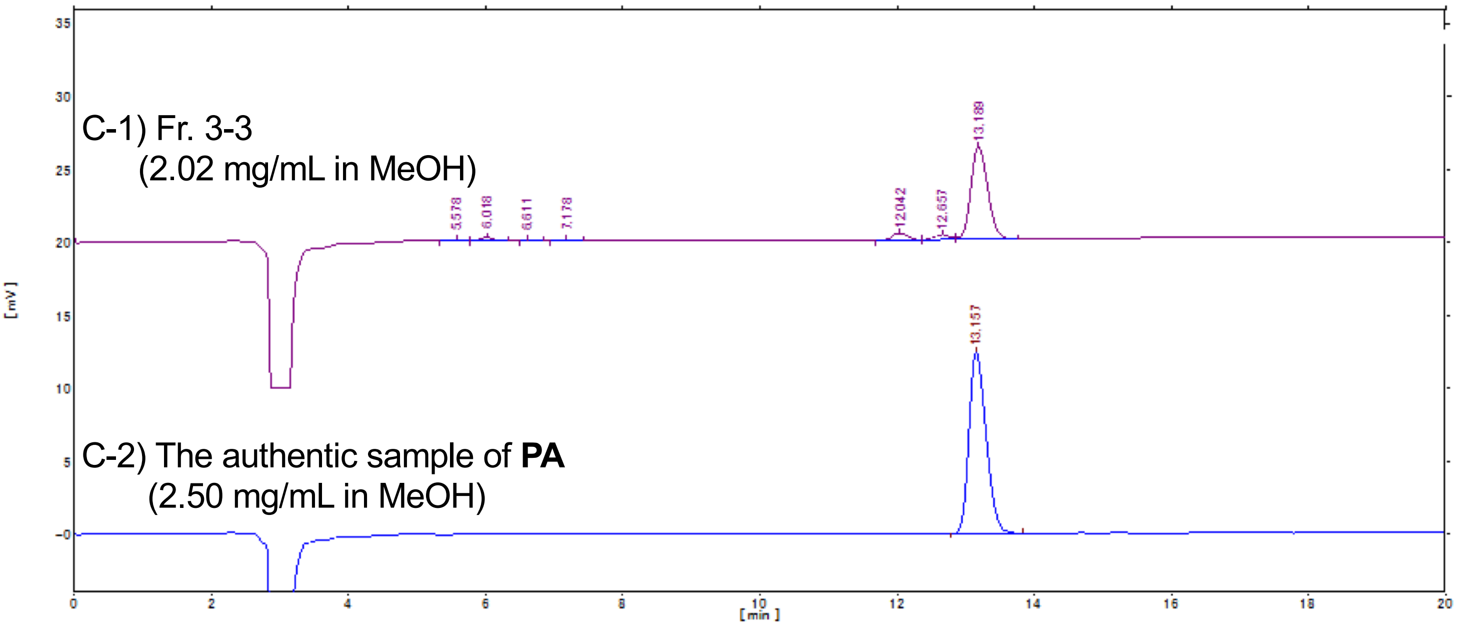

Supplementary Figure 9. Identification of PA from Fr. 3-3

(C) HPLC analysis of Fr. 3-3 compared with the authentic sample of PA.

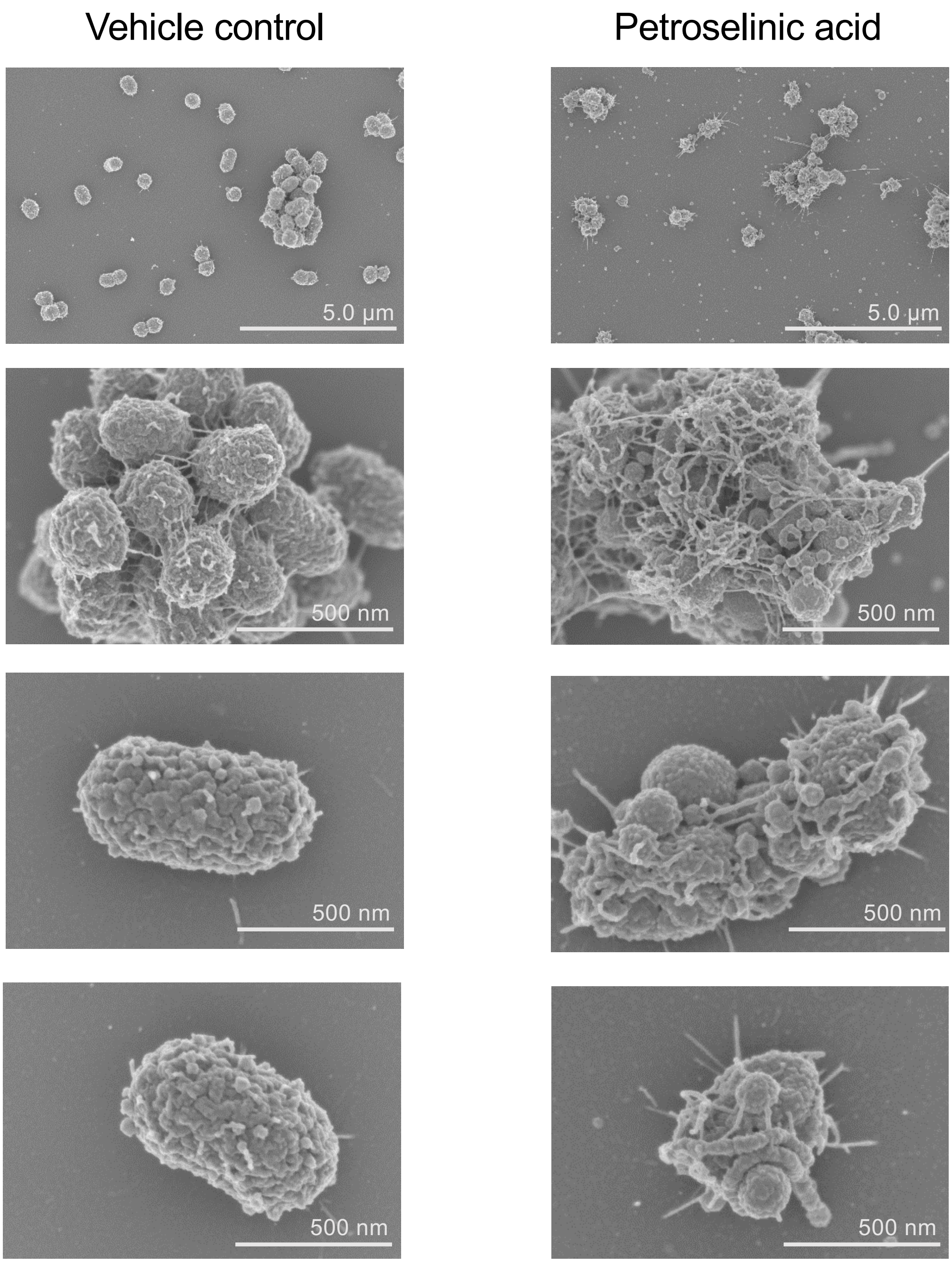

Supplementary Figure 10. Morphological observation of *P. gingivalis* ATCC 33277 treated with PA.

Bacterial cells were treated with PA at the final concentration 16 µg/mL for 30 min. Representative SEM images are shown.

(A) Area-1 (B) Area-2

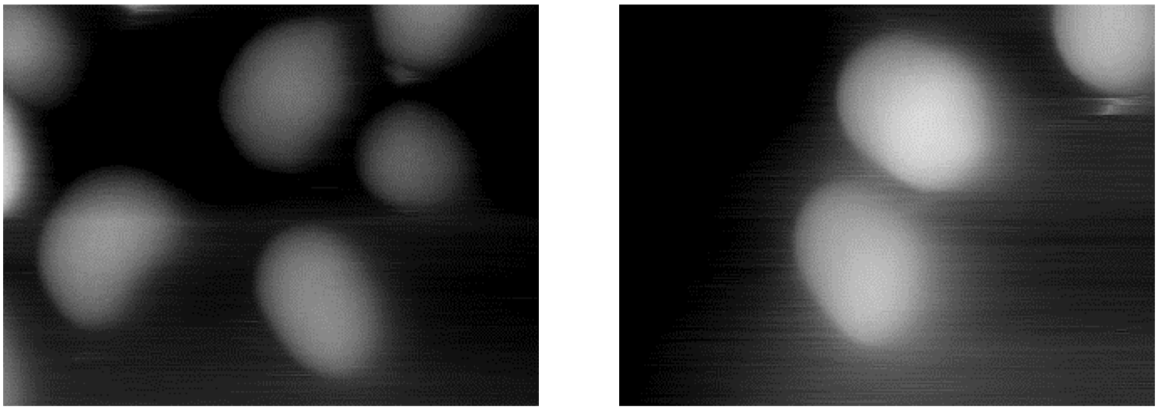

Supplementary Movie 1. Real-time bioimaging of bacterial cell surface after treatment with vehicle control.

P. gingivalis cell dynamics were monitored with a BIXAM system (Olympus). The cells were immobilized on glass slides and treated with vehicle control (1% DMSO). Images were obtained at 0.1 fps. The constructed movie is shown at 60 times the original speed. The area of each image is 3,000 × 2,250 nm^2 (x × y). Two different areas randomly chosen are shown: (A) Area-1 and (B) Area-2.

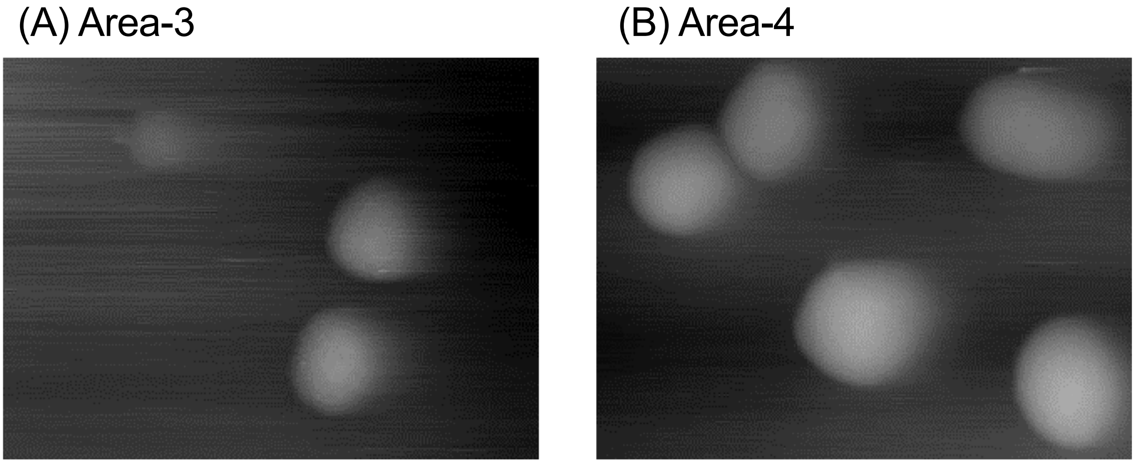

Supplementary Movie 2. Real-time bioimaging of bacterial cell surface after treatment with 46 µg/mL HEF.

P. gingivalis cell dynamics were monitored with a BIXAM system (Olympus). The cells were immobilized on glass slides and treated with HEF at 46 µg/mL. Images were obtained at 0.1 fps. The constructed movie is shown at 60 times the original speed. The area of each image is 3,000 × 2,250 nm^2 (x × y). Two different areas randomly chosen are shown: (A) Area-3 and (B) Area-4.

(A)

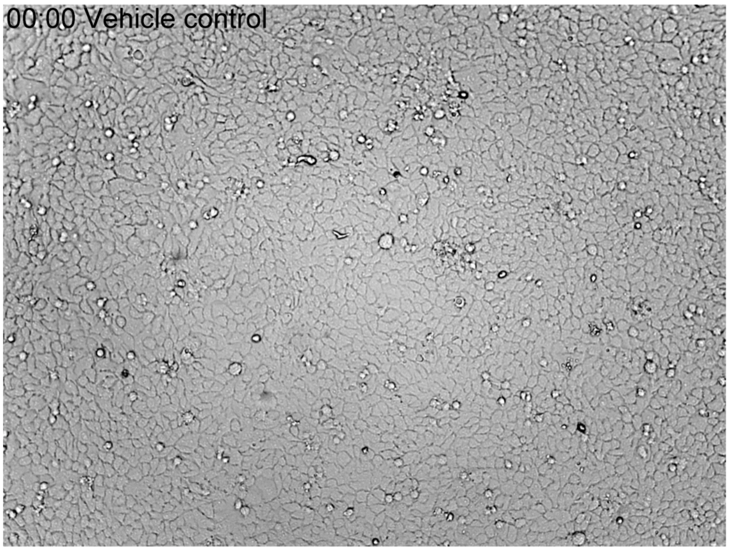

(B)

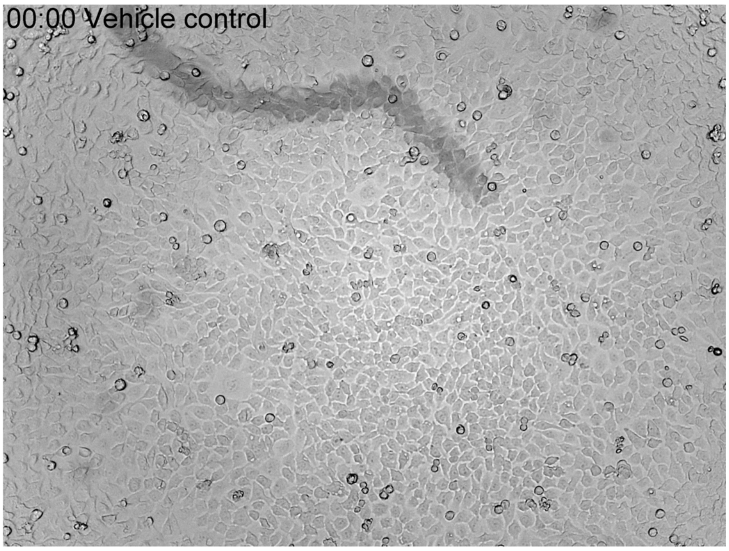

Supplementary movie 3. Time-lapse bioimaging of Ca9-22 cells after treatment with culture supernatant preincubated with vehicle control.

Images were obtained at 1 flame/min for 30 min. The movies are constructed at 320 times the original speed. Two movies from two different areas (A and B) randomly chosen are shown.

(A) (B)

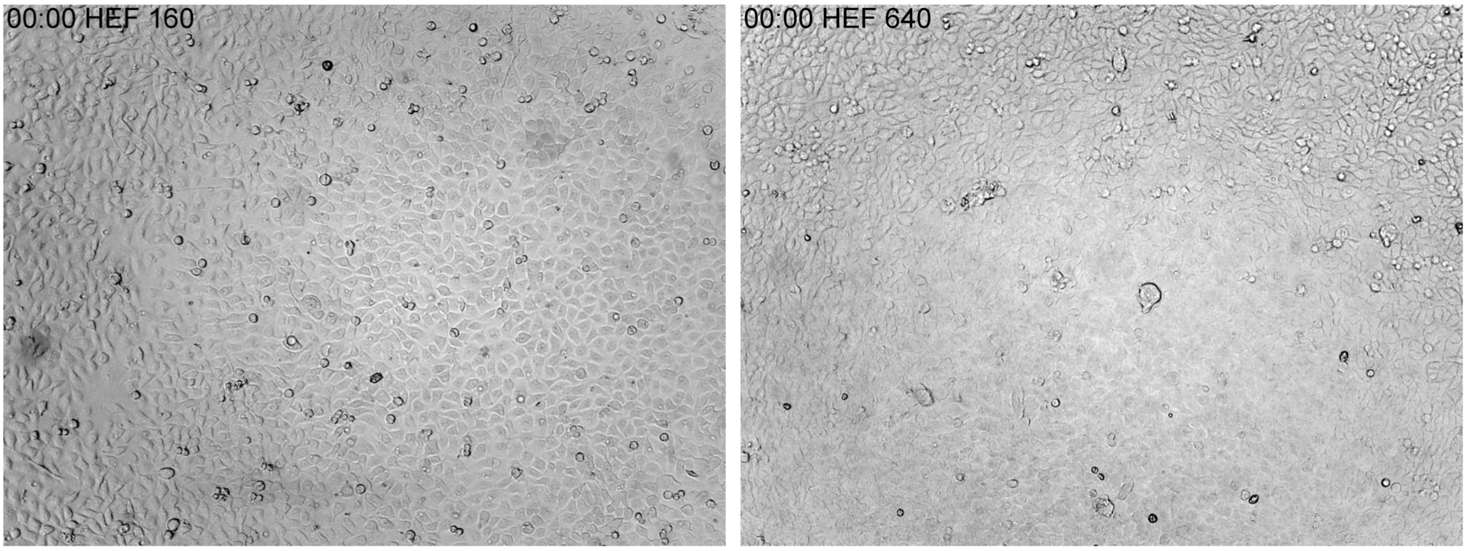

Supplementary movie 4. Time-lapse bioimaging of Ca9-22 cells after treatment with culture supernatant preincubated with HEF.

Ca9-22 cells were treated with culture supernatant preincubated with HEF at the concentrations of 160 (A) and 640 μg/mL(B). Images were obtained at 1 flame/min for 30 min. The movies are constructed at 320 times the original speed.

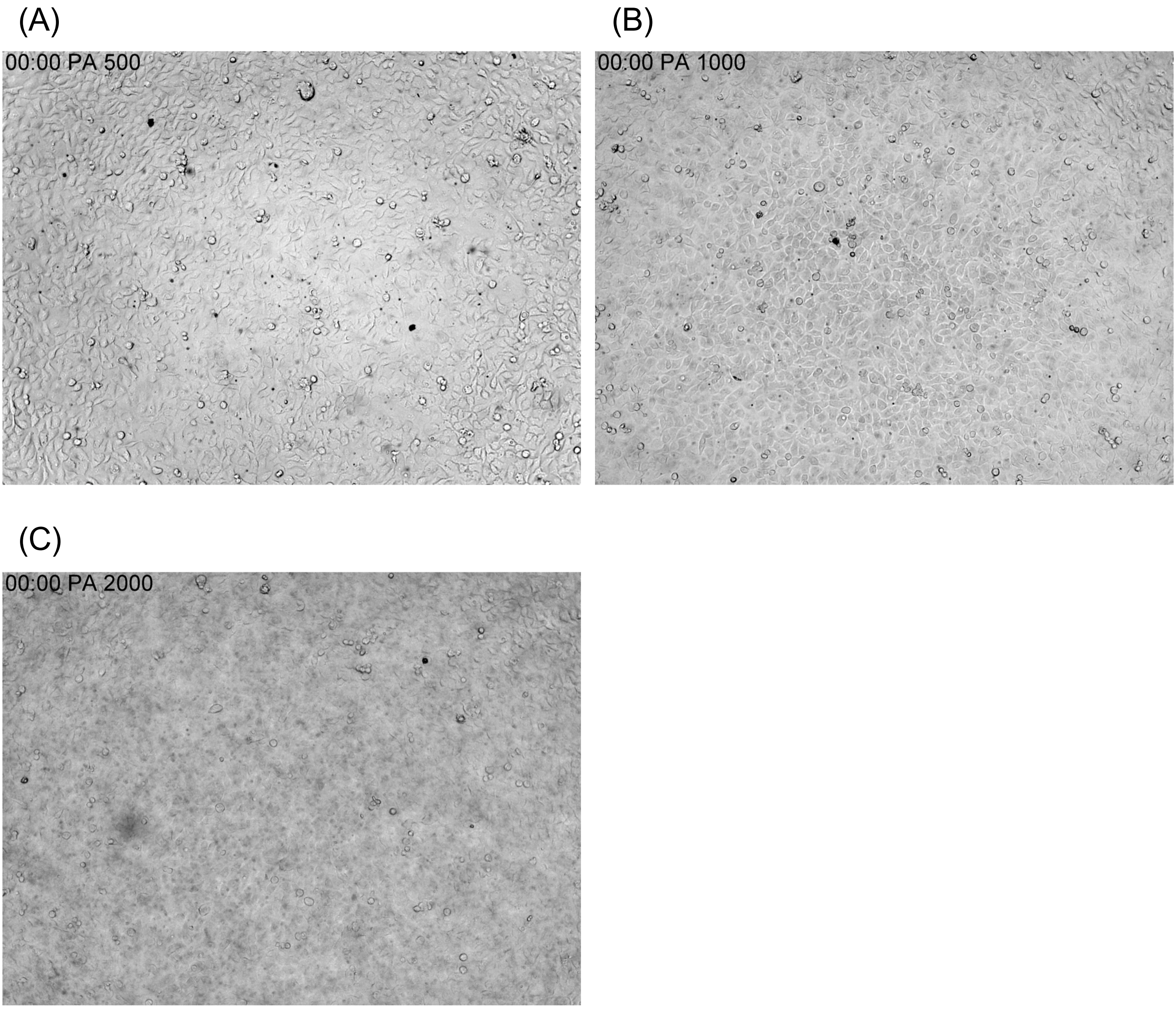

Supplementary movie 5. Time-lapse bioimaging of Ca9-22 cells after treatment with culture supernatant preincubated with PA.

Ca9-22 cells were treated with culture supernatant preincubated with PA at the concentrations of 500 (A), 1,000 µg/mL (B), and 2,000 µg/mL (C). Images were obtained at 1 flame/min for 30 min. The movies are constructed at 320 times the original speed.
